# Accuracy and cost-effectiveness of dynamic contrast-enhanced CT in the characterisation of solitary pulmonary nodules—the SPUtNIk study

**DOI:** 10.1136/bmjresp-2016-000156

**Published:** 2016-10-14

**Authors:** N R Qureshi, R C Rintoul, K A Miles, S George, S Harris, J Madden, K Cozens, L A Little, K Eichhorst, J Jones, P Moate, C McClement, L Pike, D Sinclair, W L Wong, J Shekhdar, R Eaton, A Shah, L Brindle, C Peebles, A Banerjee, S Dizdarevic, S Han, F W Poon, A M Groves, L Kurban, A J Frew, M E Callister, P Crosbie, F V Gleeson, K Karunasaagarar, O Kankam, F J Gilbert

**Affiliations:** 1 Department of Radiology, Papworth Hospital, Cambridge, UK; 2 Department of Thoracic Oncology, Papworth Hospital, Cambridge, UK; 3 Institute of Nuclear Medicine, University College London, London, UK; 4 Public Health Sciences and Medical Statistics, University of Southampton, Southampton, UK; 5 Southampton Clinical Trials Unit, University of Southampton, Southampton, UK; 6 Centre for Innovation and Leadership in Health Sciences, University of Southampton, UK; 7 Division of Imaging Sciences and Biomedical Engineering, King's College London, London, UK; 8 Department of Medical Physics, Paul Strickland Scanner Centre, Mount Vernon Hospital, East and North Herts NHS Trust, Stevenage, UK; 9 Radiation Protection Department, East and North Hertfordshire NHS Trust, Stevenage, UK; 10 Faculty of Health Sciences, University of Southampton, Southampton, UK; 11 Department of Radiology and Respiratory Medicine, Southampton University Hospitals NHS Foundation Trust, Southampton, UK; 12 Departments of Respiratory and Nuclear Medicine, Brighton and Sussex University Hospitals NHS Trust, Brighton, UK; 13 West of Scotland PET Centre, Gartnavel Hospital, Glasgow, UK; 14 Department of Radiology, Aberdeen Royal Hospitals NHS Trust, Aberdeen, UK; 15 Department of Respiratory Medicine, Leeds Teaching Hospitals NHS Trust, Leeds, UK; 16 North West Lung Centre, University Hospital of South Manchester, Manchester, UK; 17 Department of Radiology, Churchill Hospital and University of Oxford, Oxford, UK; 18 Department of Radiology, Worcestershire Royal Hospital, Worcester, UK; 19 Department of Thoracic Medicine, East Sussex Hospitals NHS Trust, Saint Leonards-on-Sea, UK; 20 Department of Radiology, University of Cambridge School of Clinical Medicine, Biomedical research centre, University of Cambridge, Cambridge, UK

**Keywords:** Lung Cancer, Imaging/CT MRI etc

## Abstract

**Introduction:**

Solitary pulmonary nodules (SPNs) are common on CT. The most cost-effective investigation algorithm is still to be determined. Dynamic contrast-enhanced CT (DCE-CT) is an established diagnostic test not widely available in the UK currently.

**Methods and analysis:**

The SPUtNIk study will assess the diagnostic accuracy, clinical utility and cost-effectiveness of DCE-CT, alongside the current CT and 18-flurodeoxyglucose-positron emission tomography) (^18^FDG-PET)-CT nodule characterisation strategies in the National Health Service (NHS). Image acquisition and data analysis for ^18^FDG-PET-CT and DCE-CT will follow a standardised protocol with central review of 10% to ensure quality assurance. Decision analytic modelling will assess the likely costs and health outcomes resulting from incorporation of DCE-CT into management strategies for patients with SPNs.

**Ethics and dissemination:**

Approval has been granted by the South West Research Ethics Committee. Ethics reference number 12/SW/0206. The results of the trial will be presented at national and international meetings and published in an Health Technology Assessment (HTA) Monograph and in peer-reviewed journals.

**Trial registration number:**

ISRCTN30784948; Pre-results.

## Background

Solitary pulmonary nodule (SPN) is defined as a discrete well-defined intraparenchymal lesion <3 cm. SPNs are present in 20–50% of individuals considered to be at high risk for lung cancer and present an important diagnostic problem. A small proportion of patients with a SPN will have early stage lung cancer with a high 5-year survival rate following surgical resection. In the National Lung Screening Trial (NLST) an SPN was identified in 24% of the 53 454 asymptomatic current or former smokers with a 30 pack year history and of these >95% were benign.

CT is the first-line investigation for SPN. Fleischner Society guidelines have been widely used for many years although recently published British Thoracic Society (BTS) guidelines using the prediction scores and volumetry are now being more widely used in the UK. In general, nodules of 8 mm or less in diameter are assessed for interval growth using serial CT surveillance. For nodules >8 mm a number of imaging options are available determined by the patient's performance status and the probability for malignancy. For patients that are potentially suitable for treatment with curative intent, positron emission tomography-CT (PET-CT) is the investigation of choice. PET-CT provides both anatomical (CT) and functional information (PET) following intravenous administration of small quantities of the radioactive glucose analogue 18-flurodeoxyglucose (^18^FDG). ^18^FDG-PET characterises SPNs on the basis of increased glucose metabolism in malignant lesions. However, false positives can occur in certain benign conditions including granulomatous disease and infective/inflammatory lesions. False-negative scans are seen in tumours with low glucose metabolism including lung adenocarcinoma with a lepidic or mucinous component and carcinoid tumours with no atypia. In order to minimise patient anxiety, risk of overdiagnosis, cumulative radiation burden and cost of performing multiple tests a streamlined diagnostic pathway that involves the least number of investigations for accurate nodule characterisation is essential. In Paul Barnett's study looking at the management of patients with SPNs for the Veterans Affairs Positron Emission Tomography Imaging Cooperative study group, the average US Medicare expenditure for clinical management of an incidental SPN was $50 233 (£30 363) when the nodule was malignant and $22 461 (£13 577) when benign.

Dynamic contrast-enhanced CT (DCE**-**CT) is an imaging technique that involves the acquisition of a series of CT images through the nodule before and at fixed time points after the injection of an iodinated contrast medium. A region of interest is placed within the nodule and the mean enhancement value (Hounsfield Units) is calculated at each time point. DCE-CT characterises SPNs on the basis of increased enhancement in malignant nodules reflecting the presence of tumour neovascularisation. Malignant nodules typically demonstrate increased contrast enhancement >15 HU compared with benign nodules.

Pooled analysis from 10 DCE-CT studies (incorporating 1167 nodules) reported a sensitivity, specificity, PPV and NPV of 93%, 76%, 80% and 95%, respectively, for differentiating malignant from benign nodules.^1^ A single study that compared the relative cost-effectiveness of DCE-CT to conventional CT surveillance and PET-based strategies for nodule evaluation, demonstrated savings of up to £2000 per patient compared with routine nodule surveillance. Furthermore, a strategy whereby patients only underwent ^18^FDG-PET if DCE-CT suggested malignancy had similar effectiveness to ^18^FDG-PET alone but was consistently less expensive.^2^

To date, only three studies have directly compared the diagnostic performances of ^18^FDG-PET and DCE-CT in the same cohort of patients. However, pooled data from these three studies evaluating 217 SPNs demonstrated that ^18^FDG-PET and DCE-CT had sensitivities of 92% and 87% and specificities of 90% and 83%, respectively.^3–5^ As yet, no large comparative multicentre trial of DCE-CT as a standalone technique or in combination with ^18^FDG-PET or integrated PET-CT for nodule characterisation has been performed.

## Study design

The SPUtNIk trial is a multicentre prospective cohort observational study designed to (1) assess the diagnostic performances of DCE-CT and ^18^FDG-PET-CT for the characterisation of SPNs in the National Health Service (NHS) setting and (2) use decision analytic modelling to assess the likely costs and health outcomes resulting from incorporation of DCE-CT into management strategies for patients with SPNs. Secondary objectives include assessment of the incremental value of incorporating the CT appearances of a SPN into the interpretation of integrated PET-CT examinations and assessing whether combining DCE-CT with ^18^FDG-PET-CT is more accurate and/or cost-effective for characterising SPNs than either test used alone or in series. The study consists of a cohort of 375 patients and the full protocol is available as an online supplementary material.

10.1136/bmjresp-2016-000156.supp1 Supplementary data

The primary inclusion criterion is the presence of a dominant indeterminate soft tissue SPN identified on CT that measures ≥8 and ≤30 mm on axial plane (with no ancillary evidence strongly indicative of malignancy) which is being considered for further evaluation with ^18^FDG-PET-CT. Potential participants will be identified either at local multidisciplinary meetings or at time of referral for investigation of a SPN. The study involves a single DCE-CT (radiation dose 25 mSv) being performed in addition to the patient's standard SPN management. The DCE-CT is performed within 21 days of the ^18^FDG-PET-CT examination. Exclusion criteria include history of malignancy within 2 years, confirmed aetiology of the SPN at the time of the qualifying CT, biopsy of the nodule before the DCE-CT, contraindication to potential surgical resection or radiotherapy and contraindication to any of the imaging investigations.

Site accreditation and quality assessment for ^18^FDG-PET-CT and DCE-CT will be performed using established procedures by the PET core laboratory at St Thomas' Hospital, London and Mount Vernon Hospital, London, respectively. The ^18^FDG-PET-CT and DCE-CT image acquisition and data analysis will follow a standardised protocol. The ^18^FDG-PET-CT images and attenuation correction CT images will initially be classified according to a five-point characterisation scale. Further quantitative analysis will consist of measurements of FDG uptake expressed as the maximum standardised uptake value. The diagnostic performance of ^18^FDG-PET will be assessed with and without incorporation of the CT appearances. The presence of incidental extrathoracic findings on PET-CT will also be recorded. DCE-CT interpretation will be performed by thoracic radiologists at each participating site. For both DCE-CT and PET-CT, central review of 10% of cases will be performed by an expert radiologist/nuclear medicine physician to ensure quality assurance.

Following PET-CT and DCE-CT examinations subsequent SPN management is determined by the local specialist lung multidisciplinary meeting. The reference standard will comprise pathological and/or imaging follow-up data at 24 months. All patients without definitive pathological findings will undergo repeat CT examinations of the chest at 3, 9 and 24 months (with or without biopsy) in accordance with Fleischner guidelines. Clinical information, including information relating to costs will be extracted using a standardised data collection form to inform the economic analysis. A subanalysis using the BTS guidelines algorithm will also be undertaken.

In parallel with the main study, a quantitative substudy (IPCARD-SPN: validated in a population of general practitioner referred chest radiograph attendees) will aim to (1) identify the positive and negative predictive values of symptoms that distinguish between malignant and non-malignant SPN and (2) ascertain whether or not the inclusion of symptoms found to distinguish between malignant and non-malignant nodules increases the diagnostic value of DCE-CT and ^18^FDG-PET-CT.

## Trial outcome measures

Primary outcome measures will include diagnostic test characteristics (sensitivity, specificity, accuracy) for ^18^FDG-PET-CT and DCE-CT in relation to a subsequent clinical diagnosis of lung cancer. The outcome measures used in the economic model will include accuracy, estimated life expectancy and quality-adjusted life years (QALYs). Costs will be estimated from an NHS perspective. Incremental cost-effectiveness ratios will compare management strategies with DCE-CT to strategies without DCE-CT, where DCE-CT is expected to cost less than half that of ^18^FDG-PET-CT.

Secondary outcome measures will include diagnostic test characteristics for ^18^FDG-PET-CT with incorporation of CT appearances and combined DCE-CT/^18^FDG-PET-CT. The incidence of incidental extrathoracic findings on ^18^FDG-PET/CT, subsequent investigations and costs will also be determined.

Recruitment will be terminated when 375 patients have been recruited and undergone DCE-CT. The study has two possible end points for each patient: either the diagnosis of lung cancer via biopsy or a diagnosis of benign or non-lung cancer via either biopsy or failure of the imaged nodule to progress (increase in size) during the 2-year follow-up period. The end of the study will be reached when the last study patient reaches either of these two end points or withdraws full consent for continuing in the study.

## Conclusions

With the potential adoption of a CT-based lung cancer screening programme in the UK, the number of patients with a SPN requiring further investigation could increase substantially. Furthermore, SPNs are a common finding on CT examinations undertaken for diagnostic or staging purposes. Novel cost-effective approaches to the assessment of SPNs will be of value to the NHS.

This study will provide accurate data on the diagnostic performances of DCE-CT and ^18^FDG-PET/CT in the NHS for the characterisation of SPNs: the decision analytic modelling will assess the likely costs and health outcomes resulting from incorporation of DCE-CT into management strategies for patients with SPNs.

## References

[1] Cronin P, Dwamena BA, Kelly AM Solitary pulmonary nodules: meta-analytic comparison of cross-sectional imaging modalities for diagnosis of malignancy. Radiology 2008;246:772–82. doi:10.1148/radiol.2463062148 18235105 10.1148/radiol.2463062148

[2] Comber LA, Keith CJ, Griffiths M Solitary pulmonary nodules: impact of quantitative contrast-enhanced CT on the cost-effectiveness of FDG-PET. Clin Radiol 2003; 58:706–11. 12943643 10.1016/s0009-9260(03)00166-1

[3] Yi CA, Lee KS, Kim BT Tissue characterization of solitary pulmonary nodule: comparative study between helical dynamic CT and integrated PET/CT. J Nucl Med 2006;47:443–50. 16513614

[4] Christensen JA, Nathan MA, Mullan BP Characterization of the solitary pulmonary nodule: 18F-FDG PET versus nodule-enhancement CT. Am J Roentgenol 2006;187: 1361–7. doi:10.2214/AJR.05.1166 17056930 10.2214/AJR.05.1166

[5] Orlacchio A, Schillaci O, Antonelli L Solitary pulmonary nodules: morphological and metabolic characterisation by FDG-PET-MDCT. Radiol Med 2007;112:157–73. doi:10.1007/s11547-007-0132-x 17361379 10.1007/s11547-007-0132-x

