# Supplementary material for: Accuracy and cost-effectiveness of dynamic contrast-enhanced CT in the characterisation of solitary pulmonary nodules—the SPUtNIk study
Source: BMJ Open Respir Res. 2016 Oct 14;3(1):e000156. doi: 10.1136/bmjresp-2016-000156 (PMC5073572; doi:10.1136/bmjresp-2016-000156)
Supplement: Supplementary data [file bmjresp-2016-000156supp.pdf]

Accuracy and Cost-Effectiveness of Dynamic Contrast Enhanced Computed Tomography in the Characterisation of Solitary Pulmonary Nodules

v 6.0 18th June 2015

SPONSOR: University Hospital Southampton NHS Foundation Trust

COORDINATING CENTRE: Southampton Clinical Trials Unit

ISRCTN no:	ISRCTN30784948
Ethics reference no:	12/SW/0206

Protocol authorised by:

**Name:**	Professor Fiona Gilbert	**Role:**	Chief Investigator
**Signature:**		**Date:**	

MAIN TRIAL CONTACT

Chief Investigator and Medical Expert: Professor Fiona Gilbert

Professor of Radiology
Department of Radiology
University of Cambridge School of
Clinical Medicine Box 218 Cambridge
Biomedical Campus Cambridge
CB2 0QQ

Tel: T 01223 746439
Email: fjg28@medschl.cam.ac.uk

TRIAL COORDINATION CENTRE

For general trial and clinical queries e.g. patient queries, trial supplies, data collection, please contact in the first instance:

SPUtNIk Clinical Trial Coordinator — Tel: 023 8120 3833

Address: Southampton Clinical Trials Unit
Southampton General Hospital
Tremona Road
SOUTHAMPTON
SO16 6YD

Tel: 023 8120 5154
Fax: 0844 774 0621
Email: ctu@southampton.co.uk
Web: www.ctu.soton.ac.uk

Registration (Mon-Fri 0900-1700) — **Tel: 023 8120 4507**
SAE / SUSAR Reporting — **Fax: 0844 774 0621**

SPONSOR

University Hospital Southampton NHS Foundation Trust is the research sponsor for this trial. For further information regarding sponsorship conditions, please contact the Director of Research and Development at:

Address: Research and Development
SGH – Level E
Laboratory and Pathology Block
SCRB MP 138
Southampton General Hospital
Tremona Road
SOUTHAMPTON
SO16 6YD

Tel: 023 8120 4901
Fax: 023 8120 8678
Web: www.uhs.nhs.uk

FUNDER

This trial is funded by NIHR Health Technology Assessment (HTA)

Protocol Information

This protocol describes the SPUtNIk trial and provides information about procedures for entering patients. The protocol should not be used as a guide for the treatment of other patients; every care was taken in its drafting, but corrections or amendments may be necessary. These will be circulated to investigators in the trial, but sites entering patients for the first time are advised to contact University of Southampton Clinical Trials Unit to confirm they have the most recent version.

Compliance

This trial will adhere to the principles outlined in the International Conference on Harmonisation Good Clinical Practice (ICH GCP) guidelines. It will be conducted in compliance with the protocol, the Data Protection Act and all other regulatory requirements, as appropriate.

TABLE OF CONTENTS

LIST OF ABBREVIATIONS 5

KEYWORDS 5

TRIAL SYNOPSIS 6

1. INTRODUCTION 10

1.1 BACKGROUND 10

1.2 RATIONALE AND RISK BENEFITS FOR CURRENT TRIAL 12

2. TRIAL OBJECTIVES 13

3. TRIAL DESIGN 13

3.1 TRIAL OUTCOME MEASURES 13

4. SELECTION AND ENROLMENT OF PATIENTS 13

4.1 SCREENING AND REGISTRATION 13

4.2 INCLUSION CRITERIA 14

4.3 EXCLUSION CRITERIA 14

5 STUDY PROCEDURES 15

5.1 BASELINE & EVALUATIVE PROCEDURES (VISIT 1, DAY 0) 15

5.2 PATIENT MANAGEMENT FOLLOWING PET-CT AND DCE-CT 15

5.4 ACCESS TO OUTCOME DATA 16

5.5 WITHDRAWAL CRITERIA 16

6. SAFETY OVERSIGHT 16

6.1 DEFINITIONS 17

6.2 CAUSALITY 17

6.3 REPORTING PROCEDURES 18

6.3.1 Pre-existing Conditions 18

6.3.2 Non serious AEs 18

6.3.4 Follow Up for AE/SAEs 19

7 DEFINITION OF END OF TRIAL 19

8 ASSESSMENT AND ANALYSIS 20

8.1 ASSESSMENT OF DIAGNOSTIC ACCURACY OF ^{18}FDG-PET/CT 20

8.2 ASSESSMENT OF DIAGNOSTIC ACCURACY OF DCE-CT: 21

8.3 ASSESSMENT OF DIAGNOSTIC ACCURACY OF COMBINED ^{18}FDG-PET AND DCE-CT: 21

8.4 REFERENCE STANDARD 21

8.5 SITE ACCREDITATION AND QUALITY CONTROL 21

8.6 SYSTEMATIC REVIEW, META-ANALYSIS AND ECONOMICS ASSESSMENT 22

8.7 ECONOMIC ANALYSIS 22

9 STATISTICS AND DATA ANALYSIS 23

9.1 STATISTICAL PLAN INCLUDING INTERIM ANALYSIS 23

10 ASSOCIATED STUDIES 25

10.1 IPCARD-SPN STUDY (APPENDICES 6-7) 25

11 REGULATORY ISSUES 26

11.1 CLINICAL TRIAL AUTHORISATION 26

11.2 ETHICS APPROVAL 26

11.3 CONSENT 26

11.4 CONFIDENTIALITY 26

11.5 INDEMNITY 26

11.6 SPONSOR 27

11.7 FUNDING 27

11.8 DEVIATIONS AND SERIOUS BREACHES 27

11.9 AUDITS AND INSPECTIONS 27

12 TRIAL MANAGEMENT 27

13 PUBLICATION POLICY 27

14 REFERENCES 28

APPENDIX 1 - TRIAL OVERSIGHT – COMMUNICATION AND RELATIONSHIP BETWEEN PARTIES 30

APPENDIX 2 - PROCEDURE FOR ^{18}FDG-PET/CT 31

APPENDIX 3 - PROCEDURE FOR DCE-CT 33

APPENDIX 4 - ACCREDITATION AND QUALITY-CONTROL OF PARTICIPATING PET-CT AND DCE-CT CENTRES 34

APPENDIX 5 - SYSTEMATIC LITERATURE REVIEW 35

APPENDIX 6 - PROTOCOL FOR IPCARD-SPN 38

APPENDIX 7 - QUESTIONNAIRE FOR IPCARD-SPN 42

LIST OF ABBREVIATIONS

AE	Adverse Event
CI	Chief Investigator
CRF	Case Report Form
CT	Computed Tomography
CTCAE	Common Terminology Criteria for Adverse Events
DCE-CT	Dynamic Contrast Enhanced – Computed Tomography
DMEC	Data Monitoring and Ethics Committee
^{18}FDG-PET	^{18}F-Fluorodeoxyglucose Positron Emission Tomography
HU	Hounsfield Units
ICH GCP	International Conference on Harmonisation of Good Clinical Practice
IMP	Investigational Medicinal Product
NCI	National Cancer Institute
NICE	National Institute for Health and Clinical Excellence
QALY	Quality Adjusted Life Year
REC	Research Ethics Committee
SAE	Serious Adverse Event
SPN	Solitary Pulmonary Nodule
TMG	Trial Management Group
TSC	Trial Steering Committee
SCTU	Southampton Clinical Trials Unit
LC	Lung Cancer
CXR	Chest X-Ray
PPV	Positive predictive value
NPV	Negative predictive value

KEYWORDS

Solitary Pulmonary Nodule (SPN), DCE-CT, ^{18}FDG-PET/CT, diagnostic, lung cancer

TRIAL SYNOPSIS

Short Title **SPUtNIk**

Long Title: Accuracy and Cost-Effectiveness of Dynamic Contrast Enhanced Computed Tomography in the Characterisation of Solitary Pulmonary Nodules

Sponsor: University Hospital Southampton NHS Foundation Trust

Sponsor Ref No: RHM RAD0030

Funder: HTA

Trial Phase: III

Indication: Lung Cancer

Primary Objectives:

- To determine with high precision, the diagnostic performances of DCE-CT and ^{18}FDG-PET/CT in the NHS for the characterisation of solitary pulmonary nodules (SPNs).
- To use decision analytic modelling to assess the likely costs and health outcomes resulting from incorporation of DCE-CT into management strategies for patients with SPNs.

Secondary Objectives:

- To assess, within an NHS setting, the incremental value of incorporating the CT appearances of a SPN into the interpretation of integrated PET/CT examinations.
- To assess whether combining DCE-CT with ^{18}FDG-PET/CT is more accurate and/or cost-effective, in the characterisation of SPNs, than either test used alone or in series.
- To document the nature and incidence of incidental extra-thoracic findings on ^{18}FDG-PET/CT and DCE-CT undertaken for the characterisation of SPNs and model their impact on cost-effectiveness.

Rationale:

A small proportion of patients with lung cancer present with a solitary pulmonary nodule (SPN) on diagnostic imaging tests. This is an important group of patients because presentation as a SPN represents early disease with high 5 year survival rates following surgical resection. However, not all SPNs are due to lung cancer and the accurate characterisation of SPNs for diagnosis of early stage lung cancer is a diagnostic challenge with significant associated health costs.

Widely adopted clinical guidelines for the subsequent investigation of SPNs recommend serial CT scans to look for subsequent growth with biopsy to confirm diagnosis. UK, National Institute for Health and Clinical Excellence (NICE) guidelines recommend ^{18}FDG-PET for the assessment of SPN in cases where a biopsy is not possible or has failed.

DCE-CT and ^{18}FDG-PET scans give different information about the SPN. Information from either scan or combined information from both scans may be better in the diagnosis of early stage lung cancer.

Trial Design: Prospective Observational

Sample size: 375

Inclusion Criteria:

- A soft tissue solitary dominant pulmonary nodule of ≥ 8mm and ≤30mm on axial plane
  - Measured on lung window using conventional CT scan
  - No other ancillary evidence strongly indicative of malignancy (e.g. distant metastases or unequivocal local invasion).
    - If clinicians and reporting radiologists believe the patient is being treated as having a single pulmonary nodule and there are other small lesions <4mm that would normally be disregarded, the patient should be included in the trial.
- Nodules already under surveillance can be included provided they have a recent or scheduled FDG-PET/CT18 years of age or over at time of providing consent
- Able and willing to consent to study

Exclusion Criteria:

- Pregnancy
- History of malignancy within the past 2 years
- Confirmed aetiology of the nodule at the time of qualifying CT scan – As this is a diagnostic study, should the aetiology of the nodule be confirmed by investigation such as FDG-PET/CT or bronchoscopy prior to consent the patient remains eligible as the intention to include is made on the analysis of the qualifying CT scan.
- Biopsy of nodule prior to DCE-CT scan
- Contra-indication to potential radiotherapy or surgery
- Contra indication to scans (assessed by local procedures)

Investigational Product: Non-CTIMP

Duration of Treatment: Non interventional trial

Concomitant Therapy: As per local practice

Control Group: Not Applicable

Primary Trial Endpoints: Primary outcome measures will include diagnostic test characteristics (sensitivity, specificity, accuracy) for ^{18}FDG-PET/CT and DCE-CT in relation to a subsequent clinical diagnosis of lung cancer. The outcome measures used in the economic model will include accuracy, estimated life expectancy, and quality adjusted life years (QALYs). Costs will be estimated from an NHS perspective. Incremental cost-effectiveness ratios will compare management strategies with DCE-CT to strategies without DCE-CT.

Secondary Trial Endpoints: Secondary outcome measures will include diagnostic test characteristics for ^{18}FDG-PET/CT with incorporation of CT appearances and combined DCE-CT/^{18}FDG-PET. The incidence of incidental extra-thoracic findings on ^{18}FDG-PET/CT and DCE-CT subsequent investigations and costs will also be determined.

Total Number of Sites: 15

SPutNIk Trial Flow Diagram

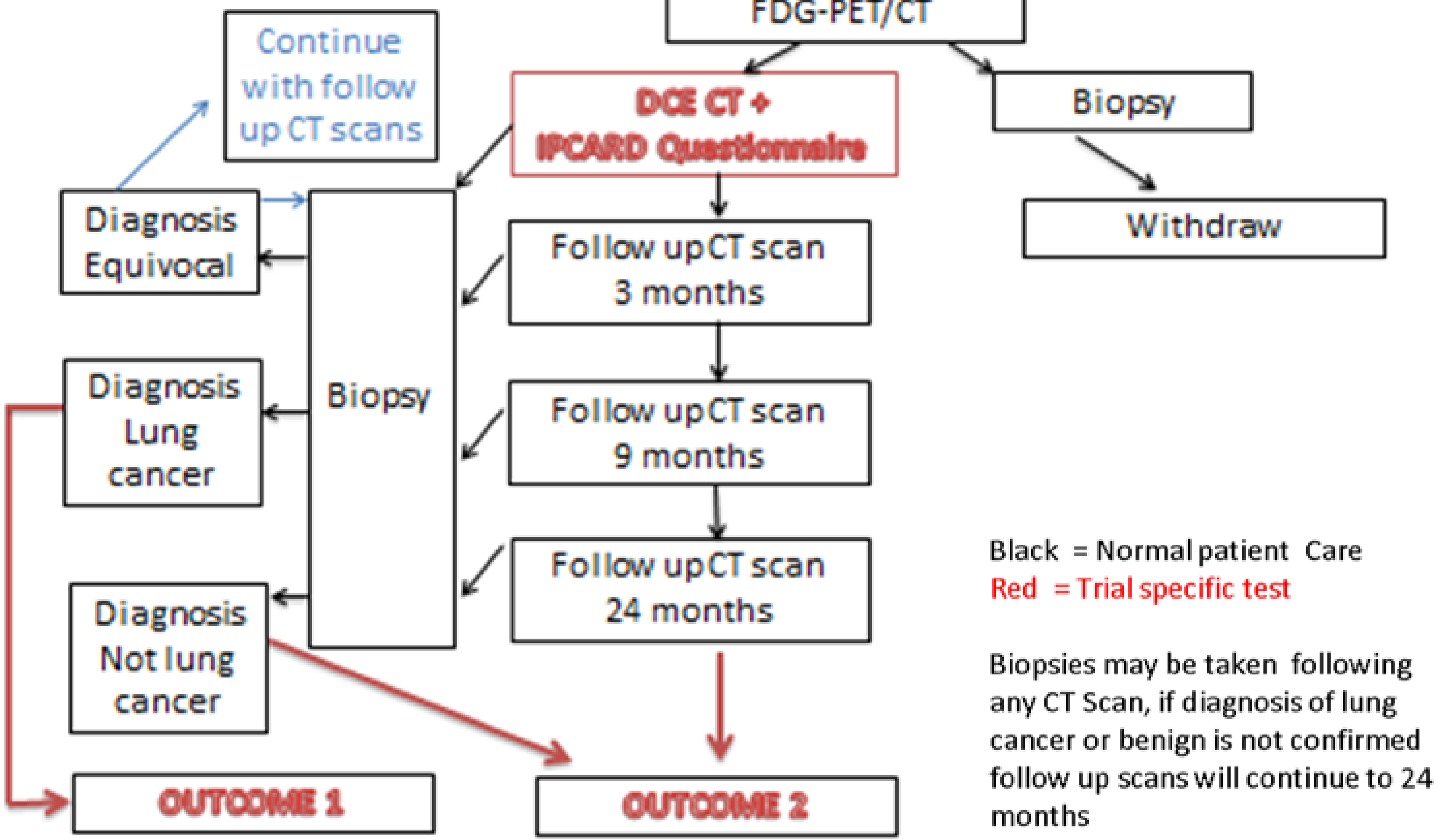

SCHEDULE OF OBSERVATIONS AND PROCEDURES

Visit:	Screening & Recruitment Visit(s)	Baseline & Diagnostics Visit 1[6]	Visit 2[7]	Visit 3[7]	Visit 4[8]	Visit 5
Day(s):	-14 to -1	0				
Week(s): Range:			3 months or local practice	9 months or local practice	12-18 months	24 months
Information Sheet provided	X					
Informed Consent		X				
Review Inclusion /Exclusion Criteria	X	X				
Recruit to Study		X				
Check contra-indications of contrast	X	X				
4-6 hr Fasting Glucose[1]		X				
Resource Assessment						X
Sub-Study IPCARD Questionnaire		X			X	
^{18}FDG-PET[1,3,5]		X				
DCE-CT[3,5]		X				
Chest CT[2,5]			X	X		X[8]
Concomitant Medications	X					
Adverse Events[4]		X				

[1] Method in accordance with standard hospital procedure for ^{18}FDG-PET.
[2] Chest CT scans will be performed only on patients without definitive pathological findings
[3] ^{18}FDG-PET and DCE-CT scans will be performed on the same day. If not possible, they should ideally be done within 14 days but no longer than 21 days.
[4] Adverse events will be recorded from DCE scan date for up to 30 days after DCE-CT scan. No effects of contrast medium are expected after 7 days
[5] Copies of Scan reports will be anonymised and sent to SCTU
[6] Baseline visit may consist of a number of actual visits, depending if PET and DCE-CT scans are performed on the same scanner and if consent is taken at scan appointment or during a separate appointment
[7]visits 2-3 will only take place for patients in whom the SPN remains undiagnosed, for patients where the scans are suggestive of lung cancer visits 2 and 3 may represent biopsy or treatment visits.
[8] this will only take place for patients in whom the SPN remains undiagnosed.

1. INTRODUCTION

1.1 BACKGROUND

Despite slow falls in the UK incidence of lung cancer, over 38,000 new cases of this disease are still diagnosed each year. Although only a small proportion of patients with lung cancer present with a solitary pulmonary nodule (SPN) on diagnostic imaging tests, this is an important group of patients because presentation as a SPN represents early disease with high 5 year survival rates following surgical resection. However, not all SPNs are due to lung cancer and the accurate characterisation of SPNs is an ongoing diagnostic challenge with significant associated health costs. A recent observational study found the average US Medicare expenditure for clinical management of a patient was $50,233 (£30,363) when the SPN was malignant and $22,461 (£13,577) when benign [1]. With the potential adoption of a Computed Tomography (CT)-based lung cancer screening programme in the UK, the number of patients with a SPN requiring further investigation could increase substantially. A previous HTA review noted that CT screening is associated with a relatively high false positive rate and subsequent investigations constitute a significant cost impact on CT screening programmes [2]. Furthermore, SPNs are a common finding in whole body screening CT examinations offered to asymptomatic individuals by independent sector providers. Typically, the costs of follow up investigations from these examinations are incurred within the public sector (UK National Screening Committee; appendix 1). Thus, novel cost-effective approaches to the assessment of SPNs would be of value to the NHS.

<u>Imaging Techniques</u>

The presence of calcification within a SPN on CT is strongly predictive of a benign cause. However, morphological features used to evaluate non-calcified SPNs by conventional CT show considerable overlap between benign and malignant nodules. Widely adopted clinical guidelines for the subsequent investigation of SPNs recommend serial CT scans for nodules of 8mm or less in diameter to look for subsequent growth [3]. The recommendation for nodules greater than 8mm is performance of dynamic Contrast Enhanced Computed Topography (DCE-CT), Fluorodeoxyglucose Positron Emission Tomography (^{18}FDG-PET) and/or biopsy, depending on local expertise [3].

In the UK, the National Institute for Health and Clinical Excellence (NICE) currently recommends ^{18}FDG-PET for the assessment of SPN in cases where a biopsy is not possible or has failed, depending on nodule size, position and CT characterisation [4]. ^{18}FDG-PET acquires images of the body following administration of small quantities of a radioactive glucose analogue. ^{18}FDG-PET characterises SPNs on the basis of increased glucose metabolism in malignant lesions. Radionuclide uptake can be assessed qualitatively or quantitatively with a Standardised Uptake Value (SUV) of greater than 2.5 implying malignancy. The most recent meta-analysis has confirmed the accuracy of ^{18}FDG-PET as a non-invasive means to characterise SPNs with pooled sensitivity 95% (95% CI: 93 -98%) and specificity 82 % (95% CI: 77 -88%) [5]. This meta-analysis included only studies using dedicated PET imaging systems because no reports on the incremental value of integrated PET-CT systems had been published at that time. In a recent audit of a local PET-CT service serving a population of 1 million with an annual lung cancer incidence of 695 patients, 44 ^{18}FDG-PET/CT scans were requested per year to characterise SPNs. Extrapolated to the UK population, the present demand for ^{18}FDG-PET to characterize SPNs is approximately 2700 scans per year, equalling almost 15% of NHS-funded PET-CT scans performed annually in the UK.

DCE-CT describes a rapid series of CT images following intravenous administration of conventional iodinated contrast media. DCE-CT characterises SPNs on the basis of increased enhancement in malignant nodules reflecting the presence of tumour neo-vascularisation. A recent meta-analysis identified 10 studies reporting the ability of DCE-CT to characterise SPNs with a pooled sensitivity of 93% (95% CI: 88% – 97%) and specificity 76% (95% CI: 68% - 97%) [5]. Due to the low cost and high sensitivity and negative predictive value of DCE-CT, this technique may be particularly valuable in the assessment of non-calcified

SPN in patients who have a low prior probability of malignancy. Currently, however, DCE-CT is not widely used in the UK.

As identified in a recent HTA review, there are no agreed guidelines for the further diagnostic investigation of SPNs identified within a CT screening programme [2]. The current NICE guidelines for the diagnosis and management of lung cancer were constructed for patients presenting symptomatically or incidentally and modifications would be required if CT screening were to be adopted in the future. The prevalence of malignancy amongst positive screenings (1.8 to 32%) is significantly lower than for SPNs presenting clinically [2]. NICE has suggested that imaging approaches may be more appropriate than biopsy for low-risk patients and therefore imaging approaches are likely to be particularly valuable for the assessment of SPNs identified within a CT screening programme. The population that would be selected for screening in the UK is as yet undetermined and is subject to an on-going HTA trial (07/82/01). It is therefore not possible to determine accurately the number of additional patients that would require further evaluation. Nevertheless, the HTA review of the clinical effectiveness and cost-effectiveness of CT screening for lung cancer concluded that a single screen at 73 years would be most cost-effective. If restricted to smokers, the most recent population statistics indicates that this would give a total UK screening population of 62,500. Based on previous publications [6,7], this population alone could yield annually approximately 5000-8000 additional non-calcified SPN's of at least 10mm in size suitable for imaging evaluation with ^{18}FDG-PET or DCE-CT. This demand would represent a significant additional burden on the currently limited availability of PET in the UK which could potentially be reduced by adoption of management strategies incorporating DCE-CT.

To date, there have been only three studies directly comparing the diagnostic performances of ^{18}FDG-PET and DCE-CT in the same cohort of patients [8-10]. Pooled data from these studies (217 SPNs) indicate ^{18}FDG-PET and DCE-CT have sensitivities of 92% and 87% and specificities of 90% and 83% respectively. As yet, no comparative studies of ^{18}FDG-PET (neither dedicated PET nor PET-CT) and DCE-CT have been performed in the UK. Therefore, in Objective 1 we will determine with high precision, the diagnostic performances of DCE-CT and ^{18}FDG-PET/CT in the NHS for the characterisation of SPNs.

<u>Therapeutic Impact and Cost-effectiveness of Imaging for SPN</u>

A single study has included an assessment of the therapeutic impact of ^{18}FDG-PET in the characterisation of SPNs [11]. This study found that ^{18}FDG-PET either contributed or was very important in reaching management decisions in 31 of 112 cases (28%). There have been several studies evaluating the cost-effectiveness of management strategies that include ^{18}FDG-PET for the characterisation of SPNs in comparison to conventional CT-based and watch and wait strategies [12-17]. These studies indicate ^{18}FDG-PET to be either cost-saving or cost-effective across several health care systems for a wide range of prior probabilities of malignancy. A range of effectiveness measures have been adopted in these studies including accuracy of management, life expectancy and in one case quality-adjusted life expectancy. In general strategies with and without ^{18}FDG-PET have demonstrated similar effectiveness but with significant differences in cost. However, these studies used neither diagnostic performance data derived from integrated PET-CT systems nor NHS cost structures.

A single study has compared the cost effectiveness of strategies that include DCE-CT to conventional CT- and PET-based strategies [18]. DCE-CT was found to offer a potentially cost-effective diagnostic approach with savings of up to £2000 per patient compared to conventional CT-based strategies. Furthermore, a strategy in which patients only underwent ^{18}FDG-PET if DCE-CT was positive for malignancy was consistently less expensive but with similar effectiveness as compared to an ^{18}FDG-PET based strategy. The cost benefits of DCE-CT were greatest when the prevalence of malignancy was low and therefore this approach may be particularly advantageous in the evaluation of SPNs found during CT screening. However, the analysis in this study was limited by the lack of direct comparative diagnostic accuracy data for DCE-CT and integrated ^{18}FDG-PET/CT at the time of writing, as well as the omission of ultimate outcome measures. Using the diagnostic performance data obtained in Objective 1, we will undertake

decision analytic modelling to assess the likely costs and health outcomes resulting from incorporation of DCE-CT into management strategies for patients with SPNs (Objective 2).

Incremental Value of incorporating CT appearances of a SPN into the interpretation of integrated PET/CT

Previous economic evaluations of ^{18}FDG-PET for SPNs have been based on diagnostic performance data for dedicated PET systems rather than integrated PET/CT. Two recent studies have shown a small incremental improvement in diagnostic performance for ^{18}FDG-PET/CT compared to ^{18}FDG-PET alone in the characterisation of SPNs [19,20]. Incorporating the CT appearances of the nodule into the diagnostic interpretation reduced the false positive rate for SPNs with moderate FDG uptake, thereby improving diagnostic specificity from 71% to 77% and 82% to 89% for the respective studies. This incremental improvement in diagnostic accuracy has the potential to impact on the cost-effectiveness of PET/CT but as yet has not been demonstrated within an NHS setting. Therefore a secondary objective of this study is to assess, within an NHS setting, the incremental value of incorporating the CT appearances of a SPN into the interpretation of integrated PET/CT examinations.

Combined DCE-CT/^{18}FDG-PET

Current integrated PET-CT systems allow for performance of ^{18}FDG-PET and DCE-CT in a single examination [21]. None of the currently published studies comparing these techniques in the assessment of SPNs has proposed diagnostic criteria that combine features from both modalities and discrepant cases are poorly reported. It is feasible that combined parameters of FDG uptake and contrast enhancement could improve the diagnostic performance of ^{18}FDG-PET by discriminating benign and malignant nodules with mildly increased FDG uptake (i.e. SUV 2.5 – 4.9). From the little data currently available, inflammatory nodules with moderate FDG uptake would be likely to exhibit higher FDG uptake/contrast enhancement ratios than malignant nodules. Furthermore, the negative predictive value of a benign result on both ^{18}FDG-PET and DCE-CT potentially could be sufficiently strong to reduce the need for subsequent imaging surveillance. Thus, a further secondary objective of this study is to assess whether combining DCE-CT with ^{18}FDG-PET/CT is more accurate and/or cost-effective in the characterisation of SPNs than either test used alone or in series.

Incidental Extra-thoracic Imaging Findings

Incidental extra-thoracic findings are not uncommon on both PET and CT components of ^{18}FDG-PET/CT examinations performed for thoracic malignancy [22]. These incidental abnormalities have the potential to add to the health outcomes and cost implications of the utilisation of ^{18}FDG-PET/CT in the characterisation of pulmonary nodules but would remain undetected by DCE-CT for which image acquisition is limited to the nodule itself. To date, economic evaluations of ^{18}FDG-PET in the characterisation of SPNs have not included this potential impact. Therefore, an additional secondary objective of this study is to document the nature and incidence of incidental extra-thoracic findings on ^{18}FDG-PET/CT undertaken for the characterisation of SPNs and model their impact on cost-effectiveness.

1.2 RATIONALE AND RISK BENEFITS FOR CURRENT TRIAL

The ethical issues of this study centre on the DCE-CT examination that patients will need to undergo which will be additional to current practice. To be applicable to patients identified within a future CT screening programme, the radiation dose associated with the DCE-CT will be kept as low as reasonably applicable and comparable to many other imaging tests commonly used in clinical practice. The contrast agent required for DCE-CT is widely used in clinical practice.

A patient information leaflet outlining the possible benefits and known risks of the study will be given to each patient and informed consent obtained. Patients unable to give consent will be excluded from the study. ^{18}FDG-PET and DCE-CT images and reports will be retained with the patient records.

2. TRIAL OBJECTIVES

Primary Objectives:

- To determine with high precision, the diagnostic performances of DCE-CT and ^{18}FDG-PET/CT in the NHS for the characterisation of solitary pulmonary nodules (SPNs).
- To use decision analytic modelling to assess the likely costs and health outcomes resulting from incorporation of DCE-CT into management strategies for patients with SPNs.

Secondary Objectives:

- To assess within an NHS setting, the incremental value of incorporating the CT appearances of a SPN into the interpretation of integrated PET/CT examinations.
- To assess whether combining DCE-CT with ^{18}FDG-PET/CT is more accurate and/or cost-effective, in the characterisation of SPNs, than either test used alone or in series.
- To document the nature and incidence of incidental extra-thoracic findings on ^{18}FDG-PET/CT and DCE-CT undertaken for the characterisation of SPNs and model their impact on cost-effectiveness.

3. TRIAL DESIGN

The study has been designed in accordance with the guidance for the methods of technology appraisal issued by NICE [23] and adopted by NICE in formulating their guidance for the use of PET in the staging of lung cancer.

The study is designed as a prospective observational study to assess the diagnostic performance and incremental value of DCE-CT by addition of this modality to ^{18}FDG-PET/CT in a cohort of 375 patients with solitary pulmonary nodule (SPN).

3.1 TRIAL OUTCOME MEASURES

Primary outcome measures will include diagnostic test characteristics (sensitivity, specificity, accuracy) for ^{18}FDG-PET/CT and DCE-CT in relation to a subsequent clinical diagnosis of lung cancer. The outcome measures used in the economic model will include accuracy, estimated life expectancy, and quality adjusted life years (QALYs). Costs will be estimated from an NHS perspective. Incremental cost-effectiveness ratios will compare management strategies with DCE-CT to strategies without DCE-CT.

Secondary outcome measures will include diagnostic test characteristics for ^{18}FDG-PET/CT with incorporation of CT appearances and combined DCE-CT/^{18}FDG-PET/CT. The incidence of incidental extra-thoracic findings on ^{18}FDG-PET/CT, subsequent investigations and costs will also be determined.

4. SELECTION AND ENROLMENT OF PATIENTS

4.1 SCREENING AND REGISTRATION

Patients will be identified either at local medical multi-disciplinary team (MDT) meetings or at the time of referral for investigation of SPN or at referral for ^{18}FDG-PET/CT to the PET centres on the basis of having a single dominant pulmonary nodule on CT scan with uncertain aetiology, for which they will be referred for a ^{18}FDG-PET/CT scan.

An ethically approved invitation letter may be sent to potential patients along with their FDG-PET/CT scan appointment letter inviting them to read the patient information sheet in advance of their appointment

and to contact the local research staff for further information and discussion about the trial should they wish to.

Local research and NHS staff will approach potential patients either in clinic or by phone to;

- Explain study and/or provide patient Information sheet
- Take age, sex and smoking history
- Confirm eligibility for study

Patients will be given an appointment for ^{18}FDG-PET/CT scan and either booked for DCE-CT scan on the same day or within 14 days of the ^{18}FDG-PET/CT scan appointment. (Note if there are scheduling issues scans may be up to a maximum of 21 days apart). Some sites may choose to make DCE appointment at ^{18}FDG-PET/CT scan following consent if there are constraints on scanner time. In this case the DCE scan should still be scheduled within 14 days of the ^{18}FDG-PET/CT scan.

SPUtNIk Patient information sheets and IPCARD-SPN questionnaire will be given to patients either in clinic or by post.

4.2 INCLUSION CRITERIA

- A soft tissue solitary dominant pulmonary nodule of ≥ 8mm and ≤30mm on axial plane
    - Measured on lung window using conventional CT scan
    - No other ancillary evidence strongly indicative of malignancy (e.g. distant metastases or unequivocal local invasion)
- If clinicians and reporting radiologists believe the patient is being treated as having a single pulmonary nodule and there are other small lesions <4mm that would normally be disregarded, the patient should be included in the trial.
- Nodules already under surveillance can be included provided they have a recent or scheduled FDG-PET/CT
- 18 years of age or over at time of providing consent
- Able and willing to consent to the study.

4.3 EXCLUSION CRITERIA

- Pregnancy
- History of malignancy within the past 2 years
- Confirmed aetiology of the nodule at the time of qualifying CT scan – As this is a diagnostic study, should the aetiology of the nodule be confirmed by investigation such as FDG-PET/CT or bronchoscopy prior to consent the patient remains eligible as the intention to include is made on the analysis of the qualifying CT scan.
- Biopsy of nodule before DCE-CT scan
- Contra-indication to potential radiotherapy or surgery
- Contra indication to scans (assessed by local practice)

All patients meeting inclusion criteria and none of the exclusion criteria will be eligible and recruited consecutively into the study. In giving consent they will be expected to follow the procedures described below

5 STUDY PROCEDURES

Sites will register patients into the SPUtNIk study by sending a signed registration form by Fax or email attachment to SCTU (numbers listed at front of protocol). Sites will be given a block of SPUtNIk codes. Sites will allocate trial ID codes consecutively following consent. On registration SCTU staff will check eligibility and confirm the patient study number by email. Both registration and the DCE-CT scan must not take place before informed consent is signed, however registration with SCTU may take place before or after the DCE-CT scan, as some clinics may schedule the DCE-CT scan outside of office hours.

5.1 BASELINE & EVALUATIVE PROCEDURES (Visit 1, Day 0)

Pre-Scan Tasks.
These tasks must be completed before study DCE –CT scans are performed**:**

- Informed consent checked or taken
- SPUtNIk trial code written on front of IPCARD-SPN Questionnaire and collection of completed questionnaires (for patients who have consented to sub study)
- Patients wishing to complete the IPCARD-SPN Questionnaire at home will be given a Free-Post return envelope to return the questionnaire

Scans:
- ^{18}FDG-PET/CT performed in line with procedure described in Appendix 2
- DCE-CT performed in line with procedure described in Appendix 3

If both scans are performed on the same day ^{18}FDG-PET/CT should be performed first with no waiting time required between scans. If scans are performed on separate days either scan can be performed first provided that patient consent and registration takes place before the DCE-CT scan (e.g. in the case of a delay in ^{18}FDG-PET/CT).

^{18}FDG-PET/CT and DCE- CT scans will ideally be performed within 14 days – however, an absolute maximum of 21 days is allowed between scans should sites have difficulty with scheduling.

5.2 PATIENT MANAGEMENT FOLLOWING PET-CT AND DCE-CT

Following the PET-CT and DCE-CT investigations, management of the SPN should be directed by the local/specialist lung MDT.

In many cases nodule biopsy or excision biopsy may be undertaken. Those cases shown to be due to lung cancer (or other malignancy) will be managed according to local protocols. Follow up/outcome data will be collected by CRF.

In some cases it is recognised that nodule surveillance is appropriate (with or without prior biopsy). In these cases it is recommended that follow up is performed at
- 3 months
- 9 months
- 24 months

as per Fleischner Society Guidelines (Radiology 2005;237:395) for management of indeterminate nodules >8mm. (Deviation from these time points based on clinical need is at the discretion of the MDT)

At each visit the following should be performed:
- Chest CT (low dose, thin section, unenhanced) unless MDT feels it is inappropriate
- Recording of any biopsy samples taken

- Recording of health resource use information

At 2 years

- Health resource data will be collected from patient records
- The end of study CRF will be completed in the InForm Database
- The PI will sign off the patients electronic CRF record and the database will be locked for that patient

5.3 PROCEDURE TO FOLLOW WHEN NODULE IS REDUCED OR NOT VISIBLE ON DCE–CT SCAN

Occasionally a nodule which was eligible (8-30mm) on lung window on qualifying CT scan has a reduced size or is not visible on mediastinal window some weeks later when the DCE-CT scan is to be performed. The reason for this may be that the nodule has resolved completely or reduced in size, due to it having an infection or inflammatory component. In this case the procedures below should be followed:

- If nodule is not visible on DCE-CT locating scan – Contrast should not be administrated and procedure should be aborted
- If nodule has reduced in size but is visible on DCE-CT locating scan with a minimum diameter of 8mm - Contrast should be administered and DCE-CT procedure followed
- If the nodule is between less than 8mm on the locating scan the decision to continue with contrast administration is to be made by the local radiologist who will assess if the nodule can be reliably reported.

Follow up and outcome data should be collected on all patients.

5.4 ACCESS TO OUTCOME DATA

If patients are lost to follow up within 2 years (e.g. relocation to another health authority) outcome data will be obtained via the Health and Social Care Information Centre. Consent for this will be taken during initial consenting process.

5.5 WITHDRAWAL CRITERIA

A patient can withdraw consent at any time. They will not be asked to give a reason for withdrawal of consent.

- If a patient withdraws from undergoing PET-CT or DCE-CT or both scans, but does not specifically withdraw consent to collect data from hospital notes , collection of relevant data from their hospital notes and GP contact should continue as per protocol.

- If consent is completely withdrawn, results may only be recorded on CRFs for procedures performed prior to the withdrawal of consent.

6. SAFETY OVERSIGHT

Safety oversight for this study is centered on the effects of injection of the contrast material and increased radiation from the DCE-CT scan that participants will undergo in addition to current practice.
To be applicable to patients identified within a future CT screening programme, the radiation dose associated with the DCE-CT is kept as low as reasonably applicable and comparable to many other imaging tests commonly used in clinical practice. The contrast agent required for DCE-CT is used extensively in clinical practice.

Adverse events will be recorded for 30 days following the DCE-CT study scan. It is envisaged that any adverse events due to radiation will be apparent within 7 days. Patients will remain in the scanner centre for at least 20 minutes following contrast injection to check and treat any immediate adverse effects. They will be given a card with the study details, DCE-CT and contrast used and local research contact details. Patients will be asked to show the card to any person that they seek medical advice from in the next 30 days and also to contact the research team if they feel unwell over the next 30 days.

All trial information will be kept for 15 years.

6.1 DEFINITIONS

Adverse Event (AE): any untoward medical occurrence in a patient or clinical trial subject which does not necessarily have a causal relationship with trial treatment or participation.

Serious Adverse Event (SAE) : any untoward medical occurrence or effect that at any dose:

- **Results in death**
- **Is life-threatening** – *refers to an event in which the patient was at risk of death at the time of the event; it does not refer to an event which hypothetically might have caused death if it were more severe*
- **Requires hospitalisation, or prolongation of existing inpatients' hospitalisation**
- **Results in persistent or significant disability or incapacity**
- **Is a congenital anomaly or birth defect**

Medical judgement should be exercised in deciding whether an AE is serious in other situations. Important AEs that are not immediately life-threatening or do not result in death or hospitalisation but may jeopardise the patient or may require intervention to prevent one of the other outcomes listed in the definition above, should also be considered serious.

6.2 CAUSALITY

The assignment of the causality to trial treatment of any serious event should be made by the investigator responsible for the care of the patient using the definitions in the table below.

If any doubt about the causality exists the local investigator should inform the SCTU who will notify the Chief Investigator. Other clinicians may be asked for advice in these cases.

In the case of discrepant views on causality between the investigator and others, all parties will discuss the case. In the event that no agreement is made, the Ethics Committee will be informed of both points of view.

Relationship	Description
**Unrelated**	There is no evidence of any causal relationship

**Unlikely**	There is little evidence to suggest there is a causal relationship (e.g. the event did not occur within a reasonable time after the test scan). There is another reasonable explanation for the event (e.g. the subject's clinical condition, other concomitant treatment).
**Possible**	There is some evidence to suggest a causal relationship (e.g. because the event occurs within a reasonable time after the test scan). However, the influence of other factors may have contributed to the event (e.g. the subject's clinical condition, other concomitant treatments).
**Probable**	There is evidence to suggest a causal relationship and the influence of other factors is unlikely.
**Definitely**	There is clear evidence to suggest a causal relationship and other possible contributing factors can be ruled out.

6.3 REPORTING PROCEDURES

All adverse events for 30 days following the DCE-CT study scan should be reported on the SPUtNIk database.

6.3.1 Pre-existing Conditions

A pre-existing condition should not be reported as an AE unless the condition worsens by at least one CTCAE (version 4.0) grade up to 30 days following the DCE-CT scan.

6.3.2 Non serious AEs

All adverse events should be recorded in the adverse event section of the online SPUtNIk InForm database and should be followed up until resolution.

6.3.3 Serious AEs

If an AE is considered serious (see definition in 6.1) In addition to recording the event in the database a paper SAE/SUSAR form should be completed and faxed to SCTU within 24 hours of site becoming aware. The SAE form can be completed by any member of the research team at site, but causality must be made by the Principal Investigator or delegated to the investigator responsible for the care of the patient. If the causality cannot be assigned within 24 hours the partially completed SAE form should be faxed to SCTU and refaxed when the PI or investigator responsible for the care of the patient is available to sign it.

The SAE/SUSAR form asks for nature of event, date of onset, severity, corrective therapies given, outcome and causality (i.e. unrelated, unlikely, possible, probably, definitely). The responsible investigator should assign the causality and expectedness of the event. Additional information should be provided as soon as possible if the event/reaction has not resolved at the time of reporting.

Diagnosis or progression of lung cancer or death, due to lung cancer and hospitalisations for elective treatment of a pre-existing condition do not need reporting as SAEs.

SAE/SUSAR REPORTING CONTACT DETAILS

Please email or fax a copy of the SAE/SUSAR form to SCTU within 24 hours of becoming aware of the event

Fax: 0844 774 0621 or Email: ctu@soton.ac.uk

FAO: Quality and Regulatory Team

For further assistance:

Tel: 023 8079 4138 (Mon to Fri 09.00 – 17.00)

The SCTU will notify the necessary REC of all SUSARs occurring during the trial according to the following timelines; fatal and life-threatening within 7 days of notification and non-life threatening within 15 days. All investigators will be informed of all SUSARs occurring throughout the trial.

Local investigators should report any SUSARs and /or SAEs as required by their Local Research Ethics Committee and/or Research & Development Office.

6.3.4 Follow Up for AE/SAEs

All adverse events should be followed by the investigator until resolved, the patient is lost to follow-up, or the adverse event is otherwise explained. When the investigator fills in an end of study form on the SPUtNIk online database they should check that all resolution dates have been recorded for AEs and SAEs before signing the case report form as complete in the database.

7 DEFINITION OF END OF TRIAL

This is a non-CTIMP study with all eligible patients recruited consecutively into the study and undergoing each study procedure. Recruitment will be terminated once 375 patients have been recruited and undergone a DCE-CT scan.

The study has two possible endpoints for each patient:

Either the diagnosis of lung cancer via biopsy **or** a diagnosis of benign or non- lung cancer via either biopsy (please note that in this case economic data collection will continue to be required for 2 years follow up)
Or failure of the imaged nodule to progress (increase in size) during the 2 year follow up period.

The end of the study will be reached when the last study patient reaches two years of follow up or withdraws full consent for continuing in the study.

PLEASE NOTE: *Health Resource Data will be collected for all patients over a 2 year period following the DCE-CT scan, therefore the end of study form will not be completed until this time unless patients have withdrawn full consent.*

8 ASSESSMENT AND ANALYSIS

8.1 ASSESSMENT OF DIAGNOSTIC ACCURACY OF ^{18}FDG-PET/CT

Analysis will be both qualitative and quantitative.

Qualitative analysis,
^{18}FDG-PET images and attenuation correction CT images will be classified according to a five-point scale using the guidelines in table 1.

An audit of the consistency of qualitative analysis will comprise of a central second reading of 10% of ^{18}FDG-PET/CT scans .

Quantitative analysis
Scans will be analysed and interpreted by PET-CT specialists at each centre.

Quantitative analysis of ^{18}FDG-PET/CT data will consist of measurements of FDG uptake expressed as the maximum standardised uptake value (SUV_{max}).

The presence of incidental extra-thoracic findings on PET and maximal tumour diameter as measured by CT will be recorded.

The diagnostic performance of ^{18}FDG-PET will be assessed with and without incorporation of the CT appearances using the guidelines in table 1 (secondary aim 1).

Based on previous studies [19, 20], the combined ^{18}FDG-PET/CT assessment will be classified as positive for malignancy if one of the following criteria are met;

- grade 4 on ^{18}FDG-PET,
- At least grade 3 on both modalities
- Grade 2 on PET and grade 3 or 4 on CT.

Grade	*Significance*	*^{18}FDG-PET*	*Attenuation Correction CT*
0	No evidence of malignancy	No visible uptake	Round, well defined lesion with laminated or popcorn calcification
1	Low probability of malignancy	Uptake less than mediastinal blood pool	Inflammatory features e.g. air bronchograms, enfolded lung
2	Indeterminate	Uptake comparable to mediastinal blood pool	Smooth well defined margins, uniform density
3	High probability of malignancy	Uptake greater than mediastinal blood pool	Lobulated, spiculated or irregular margins
4	Very high probability of malignancy	Evidence of distant metastases (i.e. M1 disease)	Evidence of distant metastases (i.e. M1 disease)

Table 1: Guidelines for the interpretation of ^{18}FDG-PET and Conventional CT

8.2 ASSESSMENT OF DIAGNOSTIC ACCURACY OF DCE-CT:

DCE-CT will be performed immediately after or within a maximum of 21 days either side of the clinically indicated PET/CT examination. Please note scans should ideally be performed within 14 days .

Interpretation of DCE-CT will comprise quantitative analysis performed by specialists at each participating site with central review for quality assurance. Quantitative analysis of DCE-CT data will consist of measurements of contrast enhancement.

8.3 ASSESSMENT OF DIAGNOSTIC ACCURACY OF COMBINED ^{18}FDG-PET AND DCE-CT:

Novel diagnostic criteria for combined ^{18}FDG-PET and DCE-CT will be developed (secondary aim 2) including quantitative values such as SUV_{max}/peak enhancement, SUV_{max}/SPV etc.

The accuracy of combined diagnostic criteria will be evaluated.

8.4 REFERENCE STANDARD

The reference standard will comprise pathological and/or imaging follow up data at 24 months as used widely in previous assessments of diagnostic performance in the characterisation of SPNs. (To restrict the reference standard to biopsy material would not be appropriate on grounds of ethics or bias. Ethical concerns would prevent biopsy of all SPNs, including those benign on imaging. Bias would be introduced if a clinically indicated biopsy were an inclusion criterion because the sample would not be representative of all patients with SPN.)

All patients without definitive pathological findings will undergo repeat CT examinations of the chest at 3, 9 and 24 months. Biopsy of lesions increasing in size during the follow up period will be performed if considered necessary on clinical grounds by the local care team.

Lesions showing a less than 20% increase in size during follow up will be considered benign. (This criterion is based on the reported doubling times of pulmonary malignancies which can be as long as 465 days, equivalent to a 20% increase in tumour diameter at 2 years [25].)

The patient’s clinical notes will also be reviewed at 24 months to determine how the patient has been managed after imaging and to confirm that decision tree models accurately reflect clinical practice (e.g. proportion of patients with imaging positive for malignancy undergoing fine-needle biopsy or surgical excision).

Clinical information will be extracted using a standardised data collection form including information relating to costs, such as investigative procedures received, surgical interventions, inpatient stays etc, and used to inform subsequent economic analyses.

The follow-up investigations and outcomes for incidental extra-thoracic findings on ^{18}FDG-PET/CT and DCE-CT will also be determined (secondary aim 3).

8.5 SITE ACCREDITATION AND QUALITY CONTROL

^{18}FDG-PET site accreditation and quality assessment will be performed using established procedures by the PET core lab at St. Thomas’ Hospital. (Appendix 4)

DCE-PET site accreditation and quality assessment will be performed using established procedures in Mount Vernon Hospital. (Appendix 4)

8.6 SYSTEMATIC REVIEW, META-ANALYSIS AND ECONOMICS ASSESSMENT

The primary outcome measures from the prospective observational study (described in section 3.1) are the diagnostic accuracy (sensitivity and specificity) for ^{18}FDG-PET/CT and DCE-CT in relation to subsequent clinical diagnosis of lung cancer. Since test results drive clinical management, patients with positive and negative imaging results are anticipated to face different care pathways and different outcomes (depending on whether their condition was correctly or incorrectly characterised). The systematic review and economic evaluation are intended to extend the analysis beyond an assessment of diagnostic accuracy to encompass the clinical utility and cost effectiveness of alternative strategies for diagnosing malignancy in people with solitary pulmonary nodules of greater than 8mm. This will be done in parallel to the current study and the outcomes will inform the development of the economic evaluation and be presented in the final report, through publication in peer review journals and at conferences. The systematic review process will be supported by an expert advisory group that includes relevant clinicians (e.g. chest physician, radiologist) and academics (e.g. methodologists).

Details of the systematic review protocol will be registered with PROSPERO database and can be found in Appendix 5.

8.7 ECONOMIC ANALYSIS

To evaluate the cost-effectiveness of the use of DCE-CT in the characterisation of SPNs, we will construct an economic model in accordance with current recommended best practice (23,26]. We propose a two stage format where a decision tree model will be developed to analyse the outcomes of diagnosis. The decision tree model will identify outcomes as: i) malignant SPN correctly diagnosed, ii) malignant SPN misdiagnosed as benign, iii) benign SPN correctly diagnosed, and iv) benign SPN misdiagnosed as malignant. The results of this will inform a Markov model to assess the longer term implications of diagnostic strategies. The Markov model will include health states for malignant disease at diagnosis (based on stage, i.e local, regional or distant), recurrence (in patients treated with curative intent) and disease-free states (for patients with benign SPN and those with malignant disease who undergo successful surgery) as well as an absorbing “death” state. The perspective will be the NHS and Personal Social Services (PSS). In the long-term model costs and benefits will be discounted using standard rates (3.5%) [23].

Uncertainty relating to input parameters and assumptions will be explored using sensitivity analyses (deterministic, and where appropriate feasible probabilistic). The key variables to be explored will include: estimates of sensitivity and specificity of diagnostic strategies; prior probability (i.e. prevalence) of malignancy (including estimates for patients with SPN detected by CT screening as well as by clinical presentation), costs of diagnostic strategies, cost of curative treatment, probabilities of disease progression in patients with malignant disease and health related quality of life (associated with disease states and with curative treatment).

Four diagnostic strategies will be addressed: ^{18}FDG-PET/CT alone, DCE-CT alone, selection for ^{18}FDG-PET/CT by preceding DCE-CT and simultaneous DCE-CT/^{18}FDG-PET. We will estimate a variety of outcome measures for use in the economic analysis; these will be cost per correctly characterised SPN and cost per life year gained. In addition, the identification of health-related quality of life data from the literature review will allow the outcome measure for the economic evaluation to be quality adjusted life years (QALYs) as recommended by NICE and the US Panel on Cost-effectiveness in Health and Medicine [23,27]. Where appropriate we will estimate the incremental cost-effectiveness ratio (ICER) of strategies including DCE-CT relative to the reference standard (^{18}FDG-PET/CT alone) – standard decision rules, relating to dominance or extended dominance, will be applied in calculating ICERs. Cost-effectiveness acceptability curves (CEACs) will be generated in any probabilistic sensitivity analysis, to illustrate the probability of the intervention being cost effective over a range of willingness to pay values.

Resource use data will be collected as part of the case review at 24-months. Resource use identified will be costed using appropriate local and national cost data. Another important source of data for the model will be from the results of the systematic review. There will be close collaboration between the systematic review and economic aspects of this study to ensure that data appropriate to the economic model is collected. In addition there will be close collaboration between the economic and clinical members of the team to ensure the model conforms to appropriate clinical realities. Although de novo modelling is planned, the model will be informed with reference to other relevant published economic modelling studies [12-18] and the possibility of adapting an existing published model will be considered. The model will be developed using standard software such as Microsoft Excel and Tree-Age Pro.

9 STATISTICS AND DATA ANALYSIS

9.1 STATISTICAL PLAN INCLUDING INTERIM ANALYSIS

Of the primary outcome measures in this study, the diagnostic characteristics for DCE-CT and ^{18}FDG-PET are used for sample size calculations. Use of the other outcome measures related to economic analyses is prevented by the prior need for detailed characterisation of the decision trees. We consider the sample size needed to detect particular accuracy for each test separately, and then when the tests are used in conjunction.

Sample size consideration for each test separately

Published sensitivity for ^{18}FDG-PET for the characterisation of SPN vary between 77 and 96% (pooled weighted average: 92%) and specificity varies between 76 and 100% (pooled weighted average: 90%). Published sensitivity and specificity values for DCE-CT vary between 81 and 100% (pooled weighted average: 87%) and 29 and 100% (pooled weighted average: 83%) respectively. Based on two previous UK studies, the mean prevalence of malignancy in indeterminate SPN has been reported as 68.5% [18]. At this prevalence, a sample size of 375 will produce approximately 257 malignant and 118 benign SPNs. This will give confidence limits for sensitivity and specificity of DCE-CT of 87% +/- 4.1% and 83% +/- 6.8% respectively, with sensitivity and specificity values for ^{18}FDG-PET of 92% +/- 3.3% and 90% +/- 5.4% respectively. These estimates will provide sufficiently narrow confidence limits to allow precise economic modelling based on the results. For the purposes of economic analyses, we will also consider combining our data with the meta-analysis results from our systematic review (section 8.2), where previous studies we currently know about total 217 patients who had undergone both techniques [8-10]. Recruitment rates are anticipated to be high (70%) because DCE-CT will be additional rather than an alternative to normal care, and is readily incorporated into the existing PET-CT examination. We expect to recruit the required sample size (375) in 18 months. With a 70% recruitment percentage of all meeting the inclusion criteria, in order to recruit 375 patients we need to invite 375/0.7 = 536 patients.

Sample size consideration for when both tests are used together

Consider if both tests are used together. In particular, (i) those DCE-CT –ve are classed as benign, and (ii) those DCE-CT +ve are then given the ^{18}FDG-PET test, with those ^{18}FDG-PET +ve classed as 'malignant' and those ^{18}FDG-PET –ve classed as 'benign'. The specificity of this process is the same as using ^{18}FDG-PET alone. So, the key interest is estimating the sensitivity of this joint test classification strategy compared to ^{18}FDG-PET alone. Based on previous data of 130 truly malignant tumour patients, there are 114 that give a DCE-CT +ve and ^{18}FDG-PET +ve result; this suggests the sensitivity of the joint testing procedure is 114 / 130 = 0.877. Compared to the ^{18}FDG-PET sensitivity thought to be 0.92 (as noted above), the joint testing approach is projected to reduce sensitivity by about 4%. Based on the sample size formula of Alonzo et al. [28], to detect that the joint DCE-CT followed by ^{18}FDG-PET approach has at least a 4% reduction in sensitivity compared to the ^{18}FDG-PET sensitivity of 0.92, a total sample size of 288 patients is required (including 197 with a truly malignant tumour); this calculation assumes an 80% power, 5% significance

level, and prevalence of malignancy of 0.685. Thus, by including 375 patients are per our previous sample size calculations above, our study is also powered to detect at least a 4% decrease in sensitivity for the joint testing approach.

Statistical analysis

(i) Diagnostic accuracy for 2 year follow-up

We will consider the diagnostic accuracy of positive ^{18}FDG-PET and DCE-CT, both separately and in conjunction, in relation to a diagnosis of lung cancer by 2 years. In these analyses we will only be able to use those patients for whom the presence of lung cancer by 2 years is known; i.e. those without diagnosed lung cancer who drop-out before the end of 2 years will be excluded. This is likely to be small.

Initially, the separate diagnostic performance of ^{18}FDG-PET/CT and DCE-CT will be examined using diagnostic criteria defined and over the full range of possible threshold values. At each threshold sensitivity and specificity will be estimated (with 95% confidence intervals), and the Receiver Operator Characteristic (ROC) curve will be calculated. The optimal cut-point from the range of values will be reported based on keeping the sensitivity above 90% and maximising specificity within this limitation. An alternative cut-point that provides the best trade off in sensitivity and specificity will also be reported. The sensitivity, specificity, positive predictive value (PPV) and negative predictive value (NPV) at these cut-points will then be presented with 95% confidence intervals. When translating sensitivity and specificity to PPV and NPV, we will need to assume a particular prevalence. This will be based on the prevalence in our study, and also we will report PPV and NPV for a range of other prevalence's reported in the literature (identified from our systematic review).

Secondly, the diagnostic performance of ^{18}FDG-PET/CT and DCE-CT combined will also be examined using the same techniques as above; we will class patients as 'positive' if they have both ^{18}FDG-PET and DCE-CT positive, and all others as 'negative'. Again, a range of different thresholds for both FDG and DCE will be considered.

Thirdly, a logistic regression model will be considered including both PET and CT, on their original scale, as covariates. This may increase the power and diagnostic accuracy of identifying those who will subsequently be diagnosed with lung cancer. It will provide a risk score and predicted probability of lung cancer for each individual, based on their specific test values. We will then use a cut-off value to decide when a risk score is high (such that we predict an adverse outcome) and when it is low (such that we predict a good outcome). The calibration of the model will be assessed by grouping patients into deciles ordered by predicted risk and considering the agreement between the mean predicted risk and the observed number of true lung cancer cases in each decile (sometimes referred to as the expected versus observed statistic, E/O). The derived diagnostic rule will be cross validated by comparing the classification of each patient with their actual primary outcome of confirmed lung cancer, allowing an estimate of the sensitivity and specificity of the prediction model. Then, by varying the chosen cut-off level, we will produce a receiver operating characteristic (ROC) curve summarising the sensitivity and specificity of the predictive rule across the range of cut-offs. The overall discriminatory ability will be summarised as the Area Under ROC curve (AUC) with 95% confidence interval. The most suitable cut-off level can then also be detected. The internal validity of the final model will also be assessed by the bootstrap re-sampling technique to adjust for over-optimism in the estimation of model performance due to validation in the same dataset that was used to develop the model itself.

If the above methods all show poor accuracy performance (in terms of calibration and/or discrimination), then the logistic regression model will be extended to include additional patient-level covariates (such as time from FDG injection to PET-CT scan), in addition to test results. Demographic information will be considered here as well as clinical and imaging features considered indicative of a higher likelihood of SPN malignancy than unselected patients. The performance of the model will also be evaluated at the

practice-level where possible, to ascertain whether model performance is consistent in each practice or, if not, the variability in performance across practices, and whether it can be improved by tailoring the prevalence used in each practice.

(ii) Flexible parametric analysis for lung cancer risk including those who drop-out before 2 years

To include any censored observations (i.e. those who were lost to follow-up before the end of 2 years and had not been diagnosed with lung cancer) a survival analysis will also be considered. This will account for the length of follow-up for each individual (regardless of their length of follow-up), and the exact time of their lung cancer diagnosis or censoring point. Kaplan-Meier plots will, if appropriate, be presented with log-rank tests to compare between the positive and negative diagnoses. In addition, we will use a flexible parametric survival analysis [29,30], that flexibly models the baseline hazard using restricted cubic splines, and computes hazard ratios for each test (on their continuous or dichotomised scale, as included), which are very similar to those from a Cox regression (which does not provide the baseline hazard though). Using the baseline hazard and hazard ratios from the fitted model – in a similar manner to the logistic regression approach described above – this will produce a risk score and a probability of being diagnosed with lung cancer by 2 years for individuals with a particular test values (or combination of test values). Calibration and discrimination of the model at 2 years of follow-up can then be checked back at the individual level. For example, for each patient their own predicted probability of being diagnosed with lung cancer by 2 years will be available from the model. Then, for each decile of predicted risk, the expected (E) probability of lung cancer will be compared with the observed (O) probability of lung cancer as obtained from a Kaplan-Meier curve of the patients in that decile. In each decile, the E/O statistic should be close to 1 if the performance of the model is consistently good across deciles. In other words, the calibration slope should be 1. The C statistic will also be calculated to examine discrimination. Bootstrapping and shrinkage methods will be used, if appropriate, to reduce over-optimism from developing and validating the model on the same set of observations. Finally, the performance of the model will be evaluated at the practice-level where possible, to ascertain whether model performance is consistent in each practice or, if not, the variability in performance across practices.

Interim analysis and data storage

No interim analysis is planned for this study

Data and all appropriate documentation will be stored for a minimum of 15 years after the completion of the trial, including the follow-up period.

10 ASSOCIATED STUDIES

10.1 IPCARD-SPN STUDY (APPENDICES 6-7)

The IPCARD study is a semi- validated symptom questionnaire which investigates the diagnostic potential of a range of general and respiratory patient reported symptoms in the diagnosis of lung related diseases.

- Patients will be asked to complete the IPCARD questionnaire at the start of the study following consent
- Patients who have not had a cancer diagnosis after one year of follow up will be sent a second copy of the questionnaire to Research teams at site will check patient notes within 1 week of posting a second IPCARD questionnaire to ensure that patients have not received a cancer diagnosis, are not terminally ill or have not died since consenting to the trial

11 REGULATORY ISSUES

11.1 CLINICAL TRIAL AUTHORISATION

This trial does NOT involve the testing of any Investigational Medicinal Products (IMPs) therefore approval from the Medicines and Healthcare products Regulatory Agency is not required.

11.2 ETHICS APPROVAL

The trial protocol has received the favourable opinion of a main Research Ethics Committee or Institutional Review Board (IRB) in the approved national participating countries.

The trial will be conducted in accordance with the recommendations for physicians involved in research on human subjects adopted by the 18th World Medical Assembly, Helsinki 1964 as revised and recognised by governing laws and EU Directives. Each patient's consent to participate in the trial should be obtained after a full explanation has been given of treatment options, including the conventional and generally accepted methods of treatment. The right of the patient to refuse to participate in the trial without giving reasons must be respected.

The investigator must ensure that the patient's anonymity will be maintained and that their identities are protected from unauthorised parties. On CRFs patients will not be identified by their names, but by an identification code. The investigator should keep a patient enrolment log showing codes, names and addresses.

11.3 CONSENT

Consent to enter the trial must be sought from each patient only after a full explanation has been given, an information leaflet offered and time allowed for consideration. The time allowed for consideration will usually be more than 24 hours, however where a patient is due to receive a biopsy on the same day as the ^{18}FDG-PET/CT scan and has been flagged, approached and had the study explained to them by phone less than 2 weekdays prior to ^{18}FDG-PET/CT and biopsy appointment, the patient may not receive the patient information sheet in the post before the ^{18}FDG-PET/CT appointment. In this case they will have received the patient information sheet 1 hour prior to giving consent so as not to be lost to the study. These cases are expected to be rare. Signed patient consent should be obtained. The right of the patient to refuse to participate without giving reasons must be respected. After the patient has entered the trial the clinician remains free to give any treatment at any stage. All patients are free to withdraw at any time from the trial without giving reasons and without prejudicing further treatment.

11.4 CONFIDENTIALITY

Patients' identification data will be required for the registration process. SCTU will preserve the confidentiality of patients taking part in the trial.

11.5 INDEMNITY

The sponsor of the trial is University Hospital Southampton NHS Foundation Trust. For NHS sponsored research HSG (96) 48 reference no.2 refers. If there is negligent harm during the clinical trial when the NHS body owes a duty of care to the person harmed, NHS Indemnity covers NHS staff, medical academic staff with honorary contracts, and those conducting the trial. NHS Indemnity does not offer no-fault compensation and is unable to agree in advance to pay compensation for non-negligent harm. Ex-gratia payments may be considered in the case of a claim.

11.6 SPONSOR

University Hospital Southampton NHS Foundation Trust is acting as sponsor for this trial. SCTU has been delegated duties by the Sponsor relating to: submissions to regulatory authorities, GCP and safety. Other delegated duties will be assigned to the NHS Trusts or others taking part in this trial and confirmed by means of the site clinical trial agreement.

11.7 FUNDING

The trial is funded by the National Institute for Health Research – Health Technology Assessment (NIHR-HTA).

11.8 DEVIATIONS AND SERIOUS BREACHES

Any trial protocol deviations/violations and breaches of Good Clinical Practice occurring at sites should be reported to SCTU and the local R&D Office immediately. SCTU will then advise of and/or undertake any corrective and preventative actions as required.

All serious protocol deviations/violations and serious breaches of Good Clinical Practice and /or the trial protocol will immediately be reported to the regulatory authorities.

11.9 AUDITS AND INSPECTIONS

The trial may be subject to inspection and audit by University Hospital Southampton NHS Foundation Trust, under their remit as sponsor, SCTU as the sponsor's delegate and other regulatory bodies to ensure adherence to ICH GCP, Research Governance Framework for Health and Social Care, applicable contracts/agreements and national regulations.

12 TRIAL MANAGEMENT

The Trial Management Group (TMG) is responsible for overseeing progress of the trial. The day-to-day management of the trial will be coordinated through the Southampton Clinical Trials Unit (SCTU) and oversight will be maintained by the Trial Steering Committee and the Data Monitoring and Ethics Committee (See Appendix 1)

13 PUBLICATION POLICY

All publications and presentations relating to the trial will be authorised by the Trial Management Group. Publication of the trial results will be in the name of the SPUtNIk Group, if this does not conflict with the journal's policy. If there are named authors, these will include at least the trial's Chief Investigator, Statistician and Trial Coordinator/Manager. SCTU will be listed as the managing organisation. Members of the TMG and the Data Monitoring Committee will be listed and contributors will be cited by name if published in a journal where this does not conflict with the journal's policy. Authorship of parallel studies initiated outside of the Trial Management Group will be according to the individuals involved in the project but must acknowledge the contribution of the Trial Management Group and SCTU.

14 REFERENCES

1. Barnett PG, Ananth L, Gould MK. Cost and outcomes of patients with solitary pulmonary nodules managed with Positron Emission Tomography. Chest. 2010 Jan; 137(1): 53-9
2. Black C, Bagust A, Boland A et al. The clinical effectiveness and cost-effectiveness of computed tomography screening for lung cancer: systematic reviews. Health Technol Assess. 2006 Jan; 10(3): iii-iv, ix-x, 1-90.
3. MacMahon H, Austin JH, Gamsu G et al. Guidelines for management of small pulmonary nodules detected on CT scans: a statement from the Fleischner Society. Radiology. 2005; 237: 395-400.
4. National Collaborating Centre for Acute Care, February 2005. Diagnosis and treatment of lung cancer. National Collaborating Centre for Acute Care, London. Available from www.rcseng.ac.uk
5. Cronin P, Dwamena BA, Kelly AM, Carlos RC. Solitary pulmonary nodules: meta-analytic comparison of cross-sectional imaging modalities for diagnosis of malignancy. Radiology. 2008; 246: 772-82.
6. Gohagan JK, Marcus PM, Fagerstrom RM et al; LUNG SCREENING STUDY RESEARCH GROUP. Final results of the Lung Screening Study, a randomized feasibility study of spiral CT versus chest X-ray screening for lung cancer. Lung Cancer. 2005; 47: 9-15.
7. Blanchon T, Bréchot JM, Grenier PA et al; Dépiscan Group. Baseline results of the Depiscan study: a French randomized pilot trial of lung cancer screening comparing low dose CT scan (LDCT) and chest X-ray (CXR). Lung Cancer. 2007; 58: 50-8.
8. Yi CA, Lee KS, Kim BT et al. Tissue characterization of solitary pulmonary nodule: comparative study between helical dynamic CT and integrated PET/CT. J Nucl Med. 2006; 47: 443-50.
9. Christensen JA, Nathan MA, Mullan BP et al. Characterization of the solitary pulmonary nodule: 18F-FDG PET versus nodule-enhancement CT. Am J Roentgenol. 2006; 187: 1361-7.
10. Orlacchio A, Schillaci O, Antonelli L et al. Solitary pulmonary nodules: morphological and metabolic characterisation by FDG-PET-MDCT. Radiol Med. 2007; 112: 157-73.
11. Herder GJ, Van Tinteren H, Comans EF et al. Prospective use of serial questionnaires to evaluate the therapeutic efficacy of 18F-fluorodeoxyglucose (FDG) positron emission tomography (PET) in suspected lung cancer. Thorax. 2003; 58: 47-51.
12. Gould MK, Sanders GD, Barnett PG et al. Cost-effectiveness of alternative management strategies for patients with solitary pulmonary nodules. Ann Intern Med 2003; 138: 724-735
13. Keith CJ, Miles KA, Griffiths MR et al. Solitary pulmonary nodules: accuracy and cost-effectiveness of sodium iodide FDG-PET using Australian data. Eur J Nucl Med Mol Imaging 2002; 29: 1016-23
14. Gambhir SS, Shepherd JE, Shah BD et al. Analytical decision model for the cost-effective management of solitary pulmonary nodules. J Clin Oncol 1998; 16: 2113-25
15. Kosuda S, Ichihara K, Watanabe M et al. Decision-tree sensitivity analysis for cost-effectiveness of chest 2-fluoro-2-D-[(18)F]fluorodeoxyglucose positron emission tomography in patients with pulmonary nodules (non-small cell lung carcinoma) in Japan. Chest 2000; 117: 346-53
16. Dietlein M, Weber K, Gandjour A et al. Cost-effectiveness of FDG-PET for the management of solitary pulmonary nodules: a decision analysis based on cost reimbursement in Germany. Eur J Nucl Med 2000; 27: 1441-56
17. Gugiatti A, Grimaldi A, Rossetti C et al. Economic analyses on the use of positron emission tomography for the work-up of solitary pulmonary nodules and for staging patients with nonsmall-cell-lung-cancer in Italy. Q J Nucl Med Mol Imaging. 2004; 48: 49-61
18. Comber LA, Keith CJ, Griffiths M, Miles KA Solitary pulmonary nodules: impact of quantitative contrast-enhanced CT on the cost-effectiveness of FDG-PET. Clin Radiol. 2003; 58: 706-11.
19. Pauls S, Buck AK, Halter G et al. Performance of integrated FDG-PET/CT for differentiating benign and malignant lung lesions--results from a large prospective clinical trial. Mol Imaging Biol. 2008; 10: 121-8.
20. Chang CY, Tzao C, Lee SC et al. Incremental Value of Integrated FDG-PET/CT in Evaluating Indeterminate Solitary Pulmonary Nodule for Malignancy. Mol Imaging Biol. 2010 Apr; 12(2): 204-9
21. Bisdas S, Spicer K, Rumboldt Z. Whole-tumor perfusion CT parameters and glucose metabolism measurements in head and neck squamous cell carcinomas: a pilot study using combined positron-emission tomography/CT imaging. Am J Neuroradiol. 2008; 29: 1376-81.
22. Marom EM, Bruzzi JF, Truong MT. Extrathoracic PET/CT findings in thoracic malignancies. J Thorac Imaging. 2006; 21: 154-66.

23. Guide to the methods of technology appraisal. National Institute for Health and Clinical Excellence, London, June 2008 ISBN: 1-84629-741-9
24. Miles KA, Young H, Chica SL, Esser PD. Quantitative contrast-enhanced computed tomography: is there a need for system calibration? Eur Radiol. 2007; 17: 919-26.
25. Miles KA, Griffiths MR, Fuentes MA. Standardized perfusion value: universal CT contrast enhancement scale that correlates with FDG PET in lung nodules. Radiology. 2001; 220: 548-53.
26. Phillips Z, Ginnelly L, Sculpher M, Claxton K, Golder S, Riemsma R et al. Review of guidelines for good practice in decision-analytic modelling in health technology assessment. Health Technol Assess 2004; 8(36): 1-172.
27. Siegel JE, Weinstein MC, Russell LB, Gold MR. Recommendations for reporting cost-effectiveness analyses. Panel on Cost-Effectiveness in Health and Medicine. JAMA. 1996; 276: 1339-41.
28. Alonzo TA, Pepe MS, Moskowitz CS. Sample size calculations for comparative studies of medical tests for detecting presence of disease. Stat Med 2002; 21:835-852
29. Royston, P and Parmar, MKB Flexible parametric proportional-hazards and proportional-odds models for censored survival data, with application to prognostic modelling and estimation of treatment effects. Statistics in Medicine 2002; 21 (15):2175 – 219
30. Lambert, P. C. and Roston, P. (2009). Further development of flexible parametric models for survival analysis. *The Stata Journal.* 9: 265-290.

APPENDIX 1 - TRIAL OVERSIGHT – Communication and Relationship between Parties

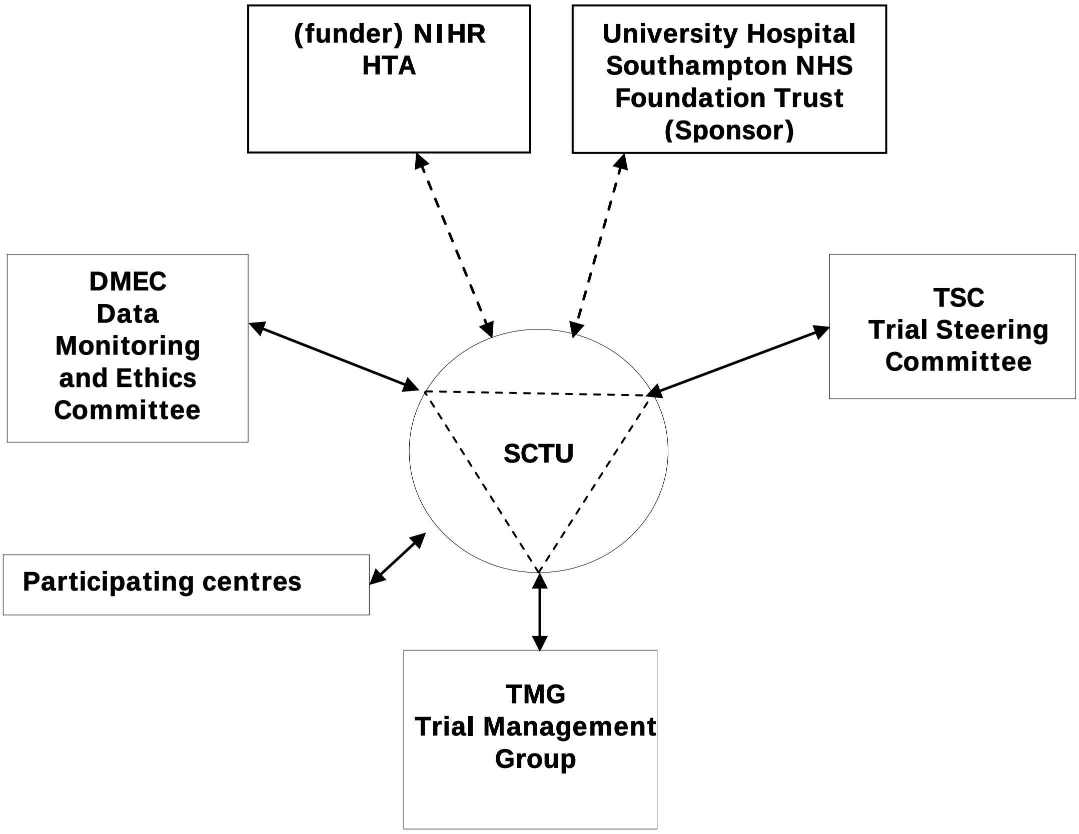

APPENDIX 2 - Procedure for ^{18}FDG-PET/CT

To maximise consistency of measurements of FDG uptake across multiple sites, we recommend that the ^{18}FDG-PET scans and examinations should follow the protocol as summarised below:
However, it is understood that for this trial ^{18}FDG-PET is standard NHS care and that in some cases the standard hospital protocol for ^{18}FDG-PET will override this protocol. In these cases we will record the differences in protocol and, where necessary take them into consideration during analysis of the trial data.

Patient Preparation and Scanning:

- Patient height and weight to be measured and recorded on arrival.
- Patients are to fast for 6 hours (4 hours for diabetics) before receiving the injection of ^{18}F-FDG. While fasting, patients should consume at least two to three 355-mL (12-oz) glasses of water to ensure adequate hydration.
- Diabetics on oral medication should be given a morning appointment, fast for 4 hours and omit their hypoglycaemic medication for the morning.
- Diabetics on insulin should eat and administer insulin as normal then fast for 6 hours prior to appointment. If blood glucose is >11mmol/l, the scan should be rescheduled (**INSULIN SHOULD NOT BE GIVEN TO LOWER BLOOD GLUCOSE LEVELS).**
- Patients should preferably avoid strenuous exercise for 6 hours before the scan to minimize uptake of the radiotracer ^{18}F-FDG in muscles.
- Blood glucose should be measured to determine confirm a concentration of less than 11 mmol/l. Insulin should not be used to adjust the blood glucose at the time of the imaging procedure.
- **Intravenous CT contrast media should not be administered prior to the ^{18}FDG-PET/CT scan. If the DCE-CT scan is to be performed on the same day it should be performed after the ^{18}FDG-PET /CT scan.**
- **A separate low dose CT without contrast should also be acquired before the PET acquisition and this scan should be used for attenuation correction of the PET images.**
- Before injection of the ^{18}F-FDG tracer, patients should urinate to minimize the possibility that they will need to move during the ^{18}F-FDG uptake phase.
- The use of all current medications should be noted. Especially those that may affect the uptake or bio-distribution of ^{18}F-FDG such as marrow stimulating cytokines or steroids.
- Patients should be placed in a comfortable position, either supine or semi-recumbent, in a dimly lit, quiet room. The room should be kept warm to avoid shivering and other temperature effects that may increase muscular or fat uptake. A large-bore intravenous line (21 gauge or greater) should be placed in an arm or hand vein.
- The injected dose of ^{18}F-FDG is dependent on the PET system used and the patient weight (based on a 70Kg individual).
    - For 2D acquisition with a minimum of 5 minutes bed position the target activity is 385MBq (+/-10%) but injected activity must not exceed 400MBq.
    - For 3D acquisitions with an overlap of 25% the minimum activity is 322MBq using 3 minutes per bed position.
    - For 3D acquisition with an overlap of 50% the minimum activity is 240MBq using 2 minutes bed position.
    - For all patients the injected activity must not exceed 400MBq.
    - For patients greater than 90Kg increased scanning time per bed position should be used rather than increase in ^{18}FDG activity to improve image quality.

- The actual injected ^{18}FDG activity must be recorded on the PET acquisition form.
- During the ^{18}FDG uptake the patient should be kept warm to avoid uptake into muscles or brown fat.
- The response scans must be performed at the same time after injection as the baseline scan± 10 minutes.
- The administration of a sedative, such as diazepam, is at the discretion of the clinician.
- Whole-body imaging should ideally begin 60 ± 10 min (mean ± SD) after injection, but this may vary according to local practice.

Image Acquisition and Reconstruction:

- Whole-body acquisitions can be in either 2- or 3-dimensional mode with attenuation correction.
- The whole-body acquisition should sample from the angle of the jaw to the level of the mid thigh.

Image Analysis:

- ^{18}FDG uptake within SPNs will be quantified as the mean and maximum Standardised Uptake Values (SUV_{max}) calculated on the basis of both body mass and body surface area.

Appendix 3 - Procedure for DCE-CT

Prior to DCE-CT, the suitability of the SPN for DCE-CT assessment will be undertaken by confirming that the nodule is visible on the low-dose CT images (acquired for attenuation correction) viewed on mediastinal windows (Window Width 400HU, Window Level 40HU).

The DCE-CT technique used has been determined by comparative measurements varying current or voltage by weight in a thorax phantom in Mount Vernon Medical Physics Department. A dynamic series of short spiral acquisitions centred on the SPN will be acquired with the patient breath-holding following an intravenous bolus of iodinated contrast material (300 mg/ml) injected intravenously at 2ml/sec. The volume of contrast material will be 1.4 ml/kg. The minimum image data set is summarised in Table 2.

Tube voltage	100 KVp,
Tube current	Determined by patients weight as follows:<br><60Kg 200mAs<br>60-90Kg 350 mAs<br>>90kg 500 mAs
Rotation time	0.5 s or similar depending on scanner
Pitch	1:1 or similar depending on scanner
Field of View	15cm or similar depending on scanner
Z-direction coverage	At least 60mm
Detector collimation	To be specified for each scanner model
Slice thickness	3.0 mm
Reconstruction interval	2.0 mm or similar depending on scanner
Image time relative to onset of contrast material injection	Pre- contrast, 60s, 120s, 180s and 240s
Reconstruction algorithm	To be specified for each scanner model. Iterative reconstruction (if available) to be switched off.

Table 2: Minimum image data set for DCE-CT.

Patient weight and volume of contrast injected must be recorded accurately.

For each time point, measurements of tumour attenuation (expressed as HU) will be determined in the axial plane from regions of interest with diameters approximately 70% of the nodule’s long- and short- axis diameters viewed on mediastinal windows Maximal nodule enhancement is determined by subtracting the baseline attenuation from each subsequent attenuation value
DCE-CT scans will be analysed and interpreted by specialists at each centre.
All DCE-CT reconstructed scan images will be transferred to Papworth Hospital where 10% will be second read for audit

Appendix 4 - Accreditation and Quality-Control of Participating PET-CT and DCE-CT centres

All PET centres will be accredited by the NCRI PET research network

Site accreditation, quality control and data transfer for PET-CT will be performed using established procedures as currently undertaken at St Thomas' hospital for other national multicentre PET-CT trials (PET-NECK & 18-30). All PET-CT data from the participating sites will be transferred to St Thomas' hospital where they will be collated and checks for data integrity and adherence to the study protocols will be performed. Images will then be transferred for subsequent audit and quality control as required.

During site validation for DCE-CT, participating centres will perform the prescribed acquisition protocol to image a phantom containing 5 solutions of contrast material at different specified concentrations as previously described by the lead applicant [33]. The relationship between iodine concentration and measured attenuation will be determined by Medical Physics at each centre for their imaging system by measuring the attenuation within each solution.

The criteria for accreditation for DCE-CT will be:

a) Successful transfer of the minimum data set of images for central review, acquired with appropriate acquisition factors, and
b) A correlation coefficient for the plot of attenuation versus iodine concentration of greater than 0.99.

During the trial, local QA of the scanner used for DCE-CT must be performed either weekly or before each study patient (whichever is most convenient for the local centre). This will consist of the following:

- Air calibration according to manufacturer's requirements
- CT number accuracy and uniformity in water phantom (owned locally)
- CT number accuracy in phantom provided for this study, containing at least two iodine contrast inserts.

Acceptable tolerance values will be provided during site validation.

Appendix 5 - Systematic Literature Review

A systematic review will be undertaken to evaluate the test accuracy, clinical utility and cost-effectiveness of ^{18}FDG-PET/CT and DCE-CT for diagnosing malignancy in people with solitary pulmonary nodules of greater than 8mm. It will adopt a broader scope than previous reviews [1], assessing different strategies for characterising SPNs (e.g. incremental value of integrated PET-CT) and their test accuracy, clinical utility and cost effectiveness. Importantly, it will provide a source of information for developing and populating the economic model. The systematic review will be undertaken in accordance with recognised guidelines [2-4]. Evidence will be identified from searches of electronic databases (from inception to current date), bibliographies of articles, grey literature sources and consultation with experts in the area. Literature searches will be undertaken by a senior information scientist. Study selection will be based on a two stage process. The full literature search results will be screened against pre-specified inclusion criteria (see Table 3). All citations that meet the criteria will be retrieved for further assessment of the full manuscripts. Data will be extracted from included studies using a pre-designed and piloted data extraction form to avoid errors.

The methodological quality of included studies will be assessed using formal tools specific to the design of the study and focusing on possible sources of bias. For diagnostic test studies, quality assessment will be conducted using the second edition of the Quality Assessment of Diagnostic Accuracy Studies (QUADAS-2) [5,6]. Quality assessment of economic evaluations will be conducted using a checklist adapted from those developed by Drummond et al [3] and Philips et al [4]. The selection of studies, extraction of data and assessment of quality will be undertaken by two reviewers, with any disagreements resolved by consensus or if necessary by arbitration involving a third reviewer.

The methods of data synthesis will be determined by the nature of the studies identified by the review, and the reported information and statistical results available therein. A narrative synthesis will summarise the results of all included studies.

Quantitative synthesis of results (i.e. meta-analysis) will be considered if there are several studies of the same design with high methodological quality. In particular, we will seek to extract 2 by 2 tables for each study that summarise the diagnostic accuracy of each test of interest; that is, the number of test positive and test negative patients who do or do not have a subsequent diagnosis of lung cancer. Meta-analyses of these 2 by 2 tables will be conducted as appropriate using the statistically robust bivariate and hierarchical methods with STATA and Cochrane Revman (v5) software.

The bivariate random-effects meta-analysis method allows the two by two tables to be appropriately pooled across studies, modelling the exact binomial distribution within studies[7], so to obtain summary (average) estimates of sensitivity and specificity, whilst accounting for the inevitable between-study heterogeneity and correlation in sensitivity and specificity caused by different study cut-off choices and other factors [8]. Subsequent to the analysis, the summary receiver operating characteristic curve, the summary positive and negative likelihood ratios, the positive and negative predictive values for particular assumed outcome prevalence's, and the summary diagnostic odds ratio can also be estimated. Importantly, 95% prediction intervals may also be calculated where data allows, providing a range for the sensitivity and specificity of each test when applied in an individual clinical setting; summary meta-analysis estimates only relate to some 'average' setting, which is not always clinically useful, but the prediction intervals translate meta-analysis back to individual practice [9].

Test accuracy may not always be reported in terms of a 2 by 2 table, instead being expressed as relative risks or odds ratios (e.g. studies examining how test results correspond to a subsequent diagnosis of lung cancer at a future time). Where reported, such measures will also be considered for extraction and synthesis using a random-effects meta-analysis model, to summarise the prognostic effect of the test.

Heterogeneity across studies will be examined using the I^2 statistic (which gives the percentage of the total variability in the data due to between-study heterogeneity) and the tau-squared statistic (which gives an estimate of the between-study variance). Where appropriate, this may involve an assessment of the effects of publication bias (using funnel plots and statistical tests), sub-group analyses or meta-regression to explore pre-specified variables [10]. Each random-effects analysis will be summarised by reporting the mean prognostic effect estimate and its confidence interval. Where possible, a 95% prediction interval will be provided for the prognostic effect in a new study, so to reveal how the effect may vary in different contexts and populations

The outcome of the systematic review and meta-analyses will inform the development of the economic evaluation and be presented in the final report, through publication in peer review journals and at conferences. The systematic review process will be supported by an expert advisory group that includes relevant clinicians (e.g. chest physician, radiologist)and academics (e.g. methodologists).

In addition to the systematic review of the strategies for characterising SPNs, a systematic literature search will be undertaken to identify relevant studies of the epidemiology and natural history of benign and malignant solitary pulmonary nodules and quality of life to inform the economic evaluation, where appropriate.

Patients	Patients under investigation for a solitary pulmonary nodule
Intervention	^{18}FDG-PET/CT and/or DCE-CT
Comparator	Resection, biopsy and/or clinical follow-up for at least 2 years
Setting	Secondary or tertiary care.
Outcome measures	Diagnostic accuracy: Diagnosis or not of lung cancer for the index test and reference standard at a given diagnostic threshold and point in time allowing calculation of sensitivity, specificity (reported or calculable) and other measures (e.g. likelihood ratio, predictive value, odds ratio and receiver operating characteristics (ROC) curve).<br><br>Diagnostic utility: change of diagnosis, tumour staging, change in clinical management and mortality.<br><br>Cost Effectiveness: costs, cost per case detected and cost per quality adjusted life year (QALY), preference-based health-related quality of life.
Design	Diagnostic accuracy: Cross-sectional studies.<br><br>Diagnostic utility: Randomised control studies, before and after studies, cohort studies (with control) or case control studies.<br><br>Cost Effectiveness: costing studies, cost effectiveness and cost utility studies.<br><br>The review will focus on the most rigorous designs.

Table 3: Inclusion criteria for systematic reviews

REFERENCES

1. Cronin P, Dwamena BA, Kelly AM, Carlos RC. Solitary pulmonary nodules: meta-analytic comparison of cross-sectional imaging modalities for diagnosis of malignancy. Radiology. 2008; 246: 772-82.
2. NHS Centre for Reviews and Dissemination. Undertaking systematic reviews of research on effectiveness: CRD guidelines for those carrying out or commissioning reviews. CRD Report 4. 2001.
3. Drummond MF, Jefferson TO. Guidelines for authors and peer reviewers of economic submissions to the BMJ. BMJ 313, 275-283. 1996.

4. Phillips Z, Ginnelly L, Sculpher M, Claxton K, Golder S, Riemsma R et al. Review of guidelines for good practice in decision-analytic modelling in health technology assessment. Health Technol Assess 2004; 8(36): 1-172.
5. Whiting P, Rutjes A, Reitsma J, Bossuyt P, Kleijnen J. The development of QUADAS: a tool for the quality assessment of studies of diagnostic accuracy included in systematic reviews. BMC Medical Research Methodology 2003; 3(1): 25.
6. Whiting PF, Rutjes AWS, Westwood ME, Mallett S, Deeks JJ, Reitsma JB, *et al*. QUADAS-2: A Revised Tool for the Quality Assessment of Diagnostic Accuracy Studies. *Annals of Internal Medicine* 2011; **155**(8):529-536
7. Hamza TH, van Houwelingen HC, Stijnen T. The binomial distribution of meta-analysis was preferred to model within-study variability. *Journal of Clinical Epidemiology* 2008; **61**(1):41-51
8. Reitsma JB, Glas AS, Rutjes AWS, Scholten RJPM, Bossuyt PM, Zwinderman AH. Bivariate analysis of sensitivity and specificity produces informative summary measures in diagnostic reviews. *Journal of Clinical Epidemiology* 2005; **58**(10):982-990.
9. Riley RD, Higgins JP, Deeks JJ. The interpretation of random-effects meta-analysis. *BMJ* 2011; 342:d549
10. Higgins JP, Thompson SG: Quantifying heterogeneity in a meta-analysis. Stat Med 21:1539-58, 2002

APPENDIX 6 - Protocol for IPCARD-SPN

BACKGROUND:

In the UK approximately 86% of those with LC are diagnosed at a stage when curative treatment is not possible and less than 25% survive one year following diagnosis [1-3]. Lung cancer is diagnosed at a later stage in the UK and survival rates are lower than in most other Western European countries [1, 3, 4]. Attempts to address late LC diagnosis in the UK have included the National Institute of Clinical Excellence (NICE) recommendation that urgent CXR is offered to patients presenting with any 1 of 10 specified symptoms [5]. However, the report acknowledges the lack of evidence about the predictive value of these symptoms and therefore the weak evidence base upon which these guidelines rest.

Recent research has strengthened evidence that LC can be symptomatic even years prior to diagnosis [6-8]. However, these studies do not provide strong evidence of the diagnostic value of early symptoms. Findings of a primary care based case-control study[1] suggest that those with LC report symptoms to their GP more frequently than randomly selected controls even 6-24 months prior to diagnosis. However the applicability of the PPVs reported for symptoms, is limited by the possibility of recording biases in GP records. Furthermore, recent research has indicated that many lung cancer symptoms are unrecognised and not reported to GPs [8].

Predictive values of symptoms for lung cancer diagnosis obtained from previous research [4, 5] and clinical decision support aids based upon these datasets (e.g. RATS [10]), do not distinguish between early and late stage lung cancer. Existing datasets based upon GP paper records do not provide the power to identify symptoms of early stage disease, and risk scores based upon these datasets assume no difference between symptoms of early and late stage LC. Electronic General Practice Research Database (GPRD) datasets, although providing greater power, do not provide detailed information about symptom experiences or record multiple symptoms with accuracy, information which is likely to be necessary to identify symptoms which predict early stage LC. The SPUtNIk study provides a unique opportunity to ascertain whether or not it is possible to distinguish between malignant and non-malignant solitary nodules on the basis of symptoms. The collection of symptom data within SPUtNIk will allow the identification of symptoms associated with early malignant disease. Apparent associations of symptoms with LC diagnosis in previous research might be explained by confounding; symptoms might be predictive of chronic respiratory disease or lung lesions more generally. The inclusion of symptom data within the SPUtNIk Trial would also enable the identification of symptoms which, when incorporated into a diagnostic algorithm might improve the diagnostic performance of ^{18}FDG-PET and DCE-CT scans. The IPCARD-SPN study will use the IPCARD symptoms risks, and co-morbidities questionnaire within a prospective study design to identify symptoms which distinguish between malignant and non-malignant solitary nodules.

IPCARD DEVELOPMENT

Qualitative research with those with lung cancer and early stage LC (RCUK fellowship) identified lay descriptions of symptom experiences in these groups [7]. These lay descriptors were incorporated into a symptom, risk and co-morbidities questionnaire which was developed with newly diagnosed LC patients (RCUK fellowship - Brindle). The questionnaire is designed to elicit information about symptom experiences or changes in health status that might have the potential to distinguish between those with LC and non-malignant disease that commonly occurs in those with a smoking history. The questionnaire contains:

- Items identified in the International Primary Care Respiratory Group guidelines designed to identify Chronic Obstructive Pulmonary Disease (COPD) and to distinguish between COPD and asthma [11].
- Lay descriptors of breathlessness identified in studies with patients with asthma, COPD, interstitial lung disease, cardiac failure and lung cancer [12].
- A component developed with LC patients that uses lay descriptors and open questions to record symptoms and changes in health in ten areas: cough, chest/shoulder pain, breathing, skin condition,

joint or bone aches, digestive and bowel changes, weight loss, haemoptysis, voice changes, hoarseness and any other changes in health in the previous two years.
- Risk information including smoking history, family history, known occupational exposures and more recently identified risk factors - previous diagnosis of pneumonia or malignancy [13]
- Co-morbidities.

The questionnaire has been validated with a CXR population (NIHR funded IPCARD Feasibility Study: co-lead by Dr Lucy Brindle and Professor Sue Wilson). This has enabled the addition of descriptors of symptoms of non-malignant chest and respiratory disease which might have discriminatory value for LC. Participants with early stage LC, late stage LC, COPD, asthma and heart disease have been purposively sampled within qualitative research to ensure that IPCARD adequately records the symptom experiences of these groups. Cognitive and semi-structured interviews have been used to establish the content validity of the questionnaire and the high quality of the symptom data obtained. Test-retest reliability will be evaluated by September 2012. In CXR populations refusal rates have been low (approximately 5%) and data completion high (>80%). Most non-response is accounted for by participants taking partially completed questionnaires away from the clinical site and not returning them by post. The average response rate for the two centres that have now completed their second episode of recruitment is >70%.

AIMS:

- To identify the PPV and NPV of symptoms that distinguish between malignant and non-malignant solitary pulmonary nodules
- To ascertain whether or not the inclusion of symptoms found to distinguish between malignant and non-malignant nodules increases the diagnostic value of DCE-CT and ^{18}FDG-PET.

SAMPLE SIZE

Sample size calculations for the analyses (assuming a two sided 5% alpha) using 257 cases of lung cancer and 118 without LC, gave 80% power to detect a difference in a rare or low frequency variable of 10% (from 5% in non-LC to 15% in LC) or 80% power to detect a difference in a more common variable of 14% (from 16% in non-LC to 30% in LC or from 22% in non-LC to 36% in LC). For a more prevalent LC symptom, 257 cases of lung cancer and 118 without LC gave 80% power to detect a difference of 16% (from 34% in non-LC to 50% in LC). The SPUtNIk Trial, which anticipates identifying 257 cases of LC and 118 non-malignant lesions, is adequately powered to detect odds ratios for all symptoms (except rare symptoms) likely to have diagnostic value (>2 or <0.5) in clinical populations.

RECRUITMENT STRATEGY AND CONSENT

Potential SPUtNIk participants will be introduced to the IPCARD-SPN study by the research nurse or PET coordinator either by phone or in clinic. They will be given the IPCARD-SPN questionnaire along with the SPUtNIk patient information sheet, which contains a section about the IPCARD-SPN study.

The questionnaire contains no identifiable patient information. Patients may fill in the questionnaire prior to attending the scan appointments and bring it along to the scan appointment. At the scan appointment the research nurse or radiographer will take consent for the SPUtNIk study, which includes an optional part for the IPCARD-SPN trial. If the patient consents to the IPCARD-SPN trial, the SPUtNIk trial code will be added to the front of the questionnaire and completed questionnaires can be left with the research nurse. For incomplete questionnaires the SPUtNIk code will be added and the patient given a Free-Post return envelope and asked to return the questionnaire by post within 2 days.

Participants will be told that they may withdraw consent at any time by telephoning the local hospital site a number is given on the patient information sheet.

Date received will be recorded for returned questionnaires and they will be prepared for automated data entry. Diagnosis will be obtained two years following recruitment from SPUtNIk Trial data.

A second copy of the IPCARD questionnaire will be posted to all patients who have not received a lung cancer diagnosis after 1 year (12-18 months) of follow up. This copy will contain the SPUtNIk code and be clearly marked IPCARD2. It will be posted with a cover letter explaining why the patient has been asked to complete the questionnaire for a second time and a prepaid envelope to return the questionnaire to SCTU.

Research staff, are instructed to ensure questionnaires are not sent to patients that have already received a diagnosis of lung cancer, are terminally ill or have died.

DATA ANALYSIS

The predictive value of symptoms for lung cancer diagnosis will be identified at 12 (12 months from recruitment/questionnaire completion) and 24 months following questionnaire completion (end of study) for the first IPCARD questionnaire, and at 12 months following questionnaire completion (2 years from recruitment/ at end of study) for the second IPCARD questionnaire.

Logistic regression will be used to obtain unadjusted and adjusted odds ratios for symptoms. The models will adjusted for age, sex and current smoking status and COPD status. COPD precedes lung cancer diagnosis in 40% to 90% of the cases and differences in prevalence of COPD between early stage and late stage lung cancer might explain differences in symptoms between these groups [14].

COPD will be defined by:

- Abnormal spirometry post-bronchodilator: defined as a forced expiratory volume in 1 second (FEV1) <70% of predicted and forced expiratory volume in 1 second/ forced vital capacity (FEV1: FVC) ratio <0.70. This is based on the American Thoracic Society standards agreed by most respiratory guidelines.

  AND/OR

- Clinical diagnosis, by the chest clinic physician, based on the presence of symptoms such as recurrent cough (productive), wheezing, dyspnoea in conjunction with pre-disposing risk factors such as smoking, age and family history.

  This data will be drawn from patients' medical records by research staff and will not be taken as part of the study

For the purposes of this study the implications of using a second definition of COPD (based upon evidence of emphysema identified by CT scan) will also be explored. It is possible that early symptoms experienced by those with resectable lung cancer might be explained by emphysema.

Age, sex and further common comorbidities which are found to differ between the LC and non-LC populations, and share symptoms with lung cancer will be included as covariates in the multivariate models. Previous research has found interactions between symptoms and risk factors to have higher PPVs than symptoms alone [6]. Items to identify individual risk of LC were included in IPCARD to determine the interaction of symptoms and established epidemiological risk factors [13], further refining estimates of individual risk in those with symptoms. Clinically plausible interactions between symptoms and interactions between symptoms and risk factors/comorbidities (pack years smoked, family history of cancer, previous malignancy, previous pneumonia, asthma, and acute respiratory infections) will be tested in the final model.

Multivariable analyses –developing a model to predict lung cancer diagnosis: A multivariable model will be developed which will be adjusted for age, sex, current smoking status, COPD status and common co-morbidities where these differ between lung cancer and non-lung cancer. Clinically plausible interactions between symptoms, and interactions between symptoms and risk factors/comorbidities (pack years smoked, family history of cancer, previous malignancy, previous pneumonia, asthma, and acute respiratory infections)

will be tested. All symptoms with an association with LC in univariate analyses will be entered into the final model.

Modelling strategy: Univariate logistic regression will be used to explore associations between each of the symptoms and the outcome variable (lung cancer). Forward stepwise multiple logistic regression analysis will be used to enter symptom variables into the analysis which were associated with lung cancer in univariate analysis ($p<0.05$). The criteria for entry into the model will be $p<=0.05$ and criteria for removal will be $p>=0.1$. To reassess variables discarded through the stepwise process, each in turn will be added to the final model and any improvement in predictive value (R^2) noted. The adjusted odds ratios for symptoms will be reported with p values. Symptoms with an independent relationship with LC will be included in a simple score (each symptom variable is given a value of 1). Analyses will calculate the sensitivity, specificity and predictive values for various levels of cut-offs to determine the optimal threshold at which the score best distinguishes between those with and without lung cancer.

REFERENCES

1. Coleman, M. P., G. Gatta, et al. (2003). "EUROCARE-3 summary: cancer survival in Europe at the end of the 20th century." Ann Oncol 14 Suppl 5: v128-49.
2. ONS (2000). "Cancer Survival in England and Wales, 1991-98." Health Statistics Quarterly 6: 71-80.
3. Richards, M. (2007). "EUROCARE-4 studies bring new data on cancer survival." Lancet Oncol 8(9): 752-3.
4. Imperatori, A., R. N. Harrison, et al. (2006). "Lung cancer in Teesside (UK) and Varese (Italy): a comparison of management and survival." Thorax 61(3): 232-9.
5. NICE. The Diagnosis and Treatment of Lung Cancer: Methods, Evidence & Guidance. 2005. NCCAC.
6. Hamilton, W., T. J. Peters, et al. (2005). "What are the clinical features of lung cancer before the diagnosis is made? A population based case-control study." Thorax 60(12): 1059-65.
7. Corner, J., J. Hopkinson, et al. (2005). "Is late diagnosis of lung cancer inevitable? Interview study of patients' recollections of symptoms before diagnosis." Thorax 60(4): 314-9.
8. Smith SM, Campbell NC, MacLeod U, Lee AJ, Raja A, Wyke S, Ziebland SB, Duff EM, Ritchie LD, Nicolson MC. Factors contributing to the time taken to consult with symptoms of lung cancer: a cross-sectional study Thorax 2009 64: 523-531
9. Jones, R., Latinovic, R, Charlton, J & Gulliford, MC. (2007) Alarm symptoms in early diagnosis of cancer in primary care: cohort study using General Practice Research Database. BMJ 2007;334:1040
10. Hamilton W et al., (2009) 'The CAPER studies: five case-control studies aimed at identifying and quantifying the risk of cancer in symptomatic primary care patients', British Journal of Cancer 101, S80-S86, Bach, P. B., J. R. Jett, et al. (2007). "Computed tomography screening and lung cancer outcomes." Jama 297(9): 953-61.
11. Levy, M. L., M. Fletcher, et al. (2006). "International Primary Care Respiratory Group (IPCRG) Guidelines: diagnosis of respiratory diseases in primary care." Prim Care Respir J 15(1): 20-34.
12. Wilcock, A., V. Crosby, et al. (2002). "Descriptors of breathlessness in patients with cancer and other cardiorespiratory diseases." J Pain Symptom Manage 23(3): 182-9.
13. Cassidy, A., J. P. Myles, et al. (2008). "The LLP risk model: an individual risk prediction model for lung cancer." Br J Cancer 98(2): 270-6.
14. Young R.P., Hopkins R.J., Christma T., Black P.N., Metcalf P., Gamble G.D. (2009) COPD prevalence is increased in lung cancer independent of age, gender and smoking history The European Respiratory Journal 34(2): 380-386

UNIVERSITY OF Southampton
School of Health Sciences

IPCARD (Identifying Symptoms that Predict Chest and Respiratory Disease)

Solitary Pulmonary Nodule Investigation

IPCARD-SPN Questionnaire

SPUtNIk code				

To be filled in by the research team

We are interested in all aspects of your health, including:

- Aspects of your health unrelated to your chest x-ray
- Your everyday health
- Any changes in your health
- Health experiences that have not made you feel unwell

Please answer questions fully even if the question does not appear relevant to your **current** health complaints.

There may be questions that do not apply to you. If this is the case you will be asked to skip to the next section or question - **you will not need to complete these sections of the form**.

Please answer the questions by shading the circle (like this ●) for the relevant option.

Section 1 - Chest and upper body aches, pain or discomfort

Q1 Have you ever experienced any discomfort in your chest, upper body or shoulders?

No ○ **Please go to Section 2, page 4** →

Yes and I still have the pain/discomfort ○ **Please go to question 3, below**

Yes but I no longer have the pain/discomfort ○ Q2 Have you had pain/discomfort in the last three months **Yes** ○ **No** ○

Please go to question 3

Q3 Please indicate whether the statements below accurately describe chest or upper body aches, pains or discomfort you have experienced **currently or within the last 3 months** by marking yes or no for each statement.

		**Yes**	**No**
a)	A niggle, pain or ache that feels like wind or indigestion but not associated with eating	○	○
b)	Discomfort or pain when laying/sitting in a particular position	○	○
c)	Discomfort or pain that feels like bruising	○	○
d)	Discomfort or pain that is not brought on by physical activity	○	○
e)	Discomfort or pain that comes and goes	○	○
f)	Discomfort or pain that feels like a muscle “pulled”	○	○
g)	Ache or pain in centre of chest or ribs	○	○
h)	Ache or pain in the side of chest or ribs	○	○
i)	Pain started in shoulder blade	○	○
j)	Pain moved round from back to front of chest	○	○

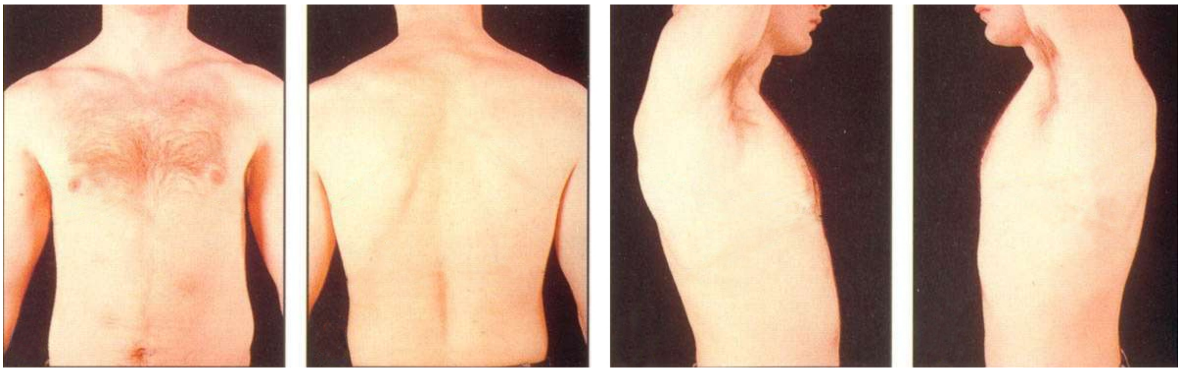

Q4 Please mark where the centre of your pain is (or pains are) with an ‘X’ on the images above.

Q5 Please indicate whether the aches, pain or discomfort described in questions 1 to 4 also occurred during the time periods below. (**Please mark all that apply**)

4-12 months ago	More than 12 months ago
O	O

Q6 In general are your aches, pain or discomfort worse than they were 3 months ago?

**Yes**		**No**	
O	**Please go to question 8**	O	**Please go to question 7**

Q7 In general are your aches, pain or discomfort worse than they were 12 months ago? **Yes** O **No** O

Q8 Please mark **one number on the scale** to indicate how much discomfort or distress the pain caused when at its worst

1	2	3	4	5	6	9	8	9	10
No discomfort/distress						Much discomfort/distress			

Section 2 - Cough

Q10 Have you ever had a cough that lasted for more than 3 weeks?

No O **Please go to page 6, section 3** →

Yes and I still have the cough O **Please go to question 12, below**

Yes but I no longer have the cough O Q11 Have you had a cough in the last three months **Yes** O **No** O

Please go to question 12

Q12 Please indicate when you **first** had a cough that lasted for more than 3 weeks.

Within the last 3 months	4-12 months ago	More than 12 months ago
O	O	O

Q13 Please indicate whether the statements below accurately describe your most recent cough/coughs (**that lasted for more than 3 weeks**) and how often you have had the type of cough described by that statement.

		Never	Once	Occasionally	Most of the time
a)	An irritating cough (feels like an irritation in the throat or chest)	○	○	○	○
b)	A tickly cough	○	○	○	○
c)	A cough that starts in the throat	○	○	○	○
d)	A cough that feels like clearing the throat	○	○	○	○
e)	A wheezy cough	○	○	○	○
f)	Cough that feels as though it arises in one or other lung or side of the chest	○	○	○	○
g)	Cough that interrupts speaking	○	○	○	○
h)	A cough without phlegm	○	○	○	○
i)	A cough that usually produces phlegm in the morning	○	○	○	○
j)	A cough that produces phlegm at any time of the day	○	○	○	○
k)	A hard or harsh cough without phlegm	○	○	○	○
l)	A hard or harsh cough that produces phlegm	○	○	○	○

Q14 Please indicate whether any of the descriptions below accurately describe a **cough that you have had within the last six months** (which has lasted for more than 3 weeks).

		**Yes**	**No**	**Not sure**
a)	Cough comes and goes	○	○	○
b)	Cough affected by the weather	○	○	○
c)	A smoker's cough	○	○	○

Q15 In general is your cough worse than it was 3 months ago?

Yes ○ **Please go to question 17**

No ○ **Please go to question 16**

Q16 In general is your cough worse than it was 12 months ago? **Yes** ○ **No** ○

Q17 Please describe any changes in your cough over time here:

Q18 Please mark **one number on the scale** to indicate how much discomfort or distress the coughing caused when at its worst

1 2 3 4 5 6 7 8 9 10

No discomfort/distress — Much discomfort/distress

Section 3 - Breathing changes

Q19 Have you ever experienced any of the following?

- becoming short of breath more easily than you used to
- unexpected shortness of breath
- noise/unusual sensation when breathing
- any difficulty breathing

No ○ **Please go to page 8, section 4** →

Yes and I still have breathing difficulties/changes ○ **Please go to question 21, below**

Yes but I no longer have breathing difficulties/changes ○ Q20 Have you had breathing difficulties/changes in the last three months? **Yes** ○ **No** ○

Please go to question 21

Q21 Please indicate when you **first** had breathing difficulties/changes

Within the last 3 months ○

4-12 months ago ○

More than 12 months ago ○

Q22 Please indicate whether the statements below accurately describe your breathing difficulties and how often you have had the type of difficulties described by that statement within the last 12 months.

		Never	Once	Occasionally	Most of the time
a)	Breathlessness after walking a short distance on the flat	○	○	○	○
b)	Breathlessness that comes on unexpectedly	○	○	○	○
c)	Breathlessness when lying down	○	○	○	○
d)	Breathlessness on resting	○	○	○	○
e)	Breathing problems that require the use of an inhaler	○	○	○	○
f)	Breathlessness that feels like an anxiety attack or that is associated with a feeling of anxiousness	○	○	○	○

Q23	Have you experienced breathing problems that are only present or get worse at certain times of the year	**Yes**	○	**No**	○

Q24 Please indicate whether any of the statements below accurately describe breathlessness that you have experienced **within the last six months** by marking yes or no for each statement:

		**Yes**	**No**
a)	Feeling out of breath	○	○
b)	Unable to get enough air	○	○
c)	Tightness in chest	○	○
d)	Breathing is shallow	○	○
e)	Breathing is rapid	○	○
f)	Feels like a weight on your chest	○	○

Q25 Please indicate whether any of the statements below accurately describe your breathing **within the last six months** by marking yes or no for each statement:

		**Yes**	**No**	**Not sure**
a)	Strange sensation felt in lung when breathing	○	○	○
b)	Wheezing noise when breathing in (for more than 2 weeks)	○	○	○
c)	Wheezing noise when breathing out (for more than 2 weeks)	○	○	○
d)	Wheezing sensation when in a particular position	○	○	○

Q26 In general is your breathlessness worse than it was 3 months ago?

**Yes**		**No**	
○	**Please go to question 28**	○	**Please go to question 27**

Q27 In general is your breathlessness worse than it was 12 months ago? **Yes** ○ **No** ○

Q28 Please mark **one number on the scale** to indicate how much discomfort or distress the change in breathing or breathlessness caused when at its worst.

1	2	3	4	5	6	7	8	9	10

No discomfort/distress — Much discomfort/distress

Section 4 - Tiredness

Q29 Have you experienced any unexpected tiredness within the last 12 months?

No ○ **Please go to Section 5, page 9** →

Yes and I still have unexpected tiredness ○ **Please go to question 31**

Yes but I no longer have unexpected tiredness ○ Q30 Have you had unexpected tiredness in the last three months? **Yes** ○ **No** ○

Please go to question 31

Q31 Please indicate when you **first** experienced any unexpected tiredness

Within the last 3 months	4-12 months ago	More than 12 months ago
○	○	○

Please answer the questions below that are all about unexpected tiredness **within the last 12 months**

		Never	Once	Occasionally	Most of the time
Q32	Have you felt tired more easily than you used to?	○	○	○	○
Q33	Have you felt as though you needed to sleep during the day?	○	○	○	○
Q34	Have you felt like you wanted to sit down or stop activity?	○	○	○	○

Q35 In general is your tiredness worse than it was 3 months ago?

**Yes**		**No**	
○	**Please go to question 37**	○	**Please go to question 36**

Q36 In general is your tiredness worse than it was 12 months ago? **Yes** ○ **No** ○

Q37	Please mark **one number on the scale** to indicate how much discomfort or distress the tiredness caused when at its worst
	1 2 3 4 5 6 7 8 9 10
	No discomfort/distress ... Much discomfort/distress

Section 5 - Coughing up Blood

Q38 Have you ever coughed up any blood?

No ○ **Please go to Section 6, page 9** →

Yes and I am still coughing up blood ○ **Please go to question 40**

Yes but I am no longer coughing up blood ○ Q39 Have you coughed up blood in the last three months? **Yes** ○ **No** ○

Please go to question 40

Q40 Please indicate when you **first** coughed up any blood

Within the last 3 months	4-12 months ago	More than 12 months ago
○	○	○

		No	Once	Occasionally	Most of the time
Q41	Have you ever coughed up mostly blood (blood with little or no phlegm)?	○	○	○	○
Q42	Have you ever coughed up phlegm with small amounts of blood?	○	○	○	○

Section 6 - Chest and respiratory infections and colds

		**Yes**	**No**
Q43	Have you currently got a phlegmy chest or chest infection?	○	○
Q44	Have you currently got a cold, flu or any other type of infection that has caused a cough or affected your breathing?	○	○

Q45 How many times have you had a chest infection within the last 12 months?

0	1	2-3	More than 3
○	○	○	○

Q46	Have you had noticeably more chest infections within the last 12 months than in the year before (13-24 months ago)?	**Yes** ○	**No** ○

Q47 How many times have you had an infection that has caused a cough or affected your breathing, a cold or flu within the last 12 months?

0	1	2-3	More than 3
○	○	○	○

Q48	Have you had noticeably more colds or flu within the last 12 months than in the year before (13-24 months ago)?	**Yes** ○	**No** ○

Section 7 - Changes in Weight

		**Yes**	**No**
Q49	Do you have to eat more than you used to in order to maintain a steady weight?	○	○
Q50	Do you now weigh less than you have for most of your adult life?	○	○
Q51	Within the last 12 months have you unintentionally lost weight that you have not regained?	○	○
Q52	Have you gained weight within the last 12 months?	○	○

Section 8 - Hot or Cold Sweats

Q53 Have you experienced hot or cold sweats during the night or day within the last two years?

**No**	○	**Please go to Section 9, page 11** →
**Yes** and I am still experiencing hot or cold sweats	○	**Please go to question 55**
**Yes** but I am no longer experiencing hot or cold sweats	○	Q54 Have you had hot or cold sweats in the last three months? **Yes** ○ **No** ○ **Please go to question 55**

Q55 Please indicate when you **first** experienced hot or cold sweats during the night or day

Within the last 3 months	4-12 months ago	More than 12 months ago
○	○	○

		Never	Once	Occasionally	Most of the time
Q56	Have you experienced hot or cold sweats in the night?	○	○	○	○
Q57	Have you experienced hot or cold sweats in the day?	○	○	○	○

		Yes	No	Not sure
Q58	Do you think all of your hot or cold sweats are probably caused by the menopause?	○	○	○

Section 9 - Eating Changes

		Yes	No
Q59	Has your appetite increased within the last 12 months?	○	○
Q60	Has your appetite decreased within the last 12 months?	○	○
Q61	Have you experienced any taste changes within the last 2 years?	○	○
Q62	Have you currently gone off certain foods you used to eat?	○	○

Section 10 - Arms, Legs and Joints

		No	Within the last 3 months	4-12 months ago	1-2 years ago
Q63	Have you experienced a **new** aching sensation in any joints or any new joint pain in the last 2 years?	○	○	○	○
Q64	Have you experienced any **new** unusual sensations or tingling in your arms or legs in the last 2 years?	○	○	○	○

Section 11 - Voice Changes

Q65 Have you experienced any **ongoing** changes in the sound of your voice when speaking?

Yes

○ **Please go to question 66**

No →

○ **Please go to Section 12, page 12**

Q66 Please indicate when you first experienced changes in the sound of your voice when speaking

Within the last 3 months	4-12 months ago	More than 12 months ago
○	○	○

Section 12 - Skin Changes

Q67 Have you experienced any changes in the condition of your skin in the last two years?

Yes

○ **Please go to question 68**

No →

○ **Please go to Section 13, page 12**

Q68 Please describe any changes in the condition of your skin.

Section 13 - Any other illnesses

Q69 Have you been told by a doctor that you had the illnesses listed below? (**Please mark one response for each condition**):

		Never	Within the last 3 months	4-12 months ago	1-5 years ago	More than 5 years ago
a)	Pneumonia	○	○	○	○	○
b)	Bronchitis or chronic bronchitis	○	○	○	○	○
c)	Asthma	○	○	○	○	○
d)	Seasonal allergy (e.g. hay fever or seasonal breathing problems).	○	○	○	○	○
e)	Chronic Obstructive Pulmonary Disease (COPD)	○	○	○	○	○
f)	Heart Disease or Angina	○	○	○	○	○

		Never	Within the last 3 months	4-12 months ago	1-5 years ago	More than 5 years ago
g)	Anaemia	○	○	○	○	○
h)	Cancer	○	○	○	○	○
i)	Emphysema or pulmonary fibrosis	○	○	○	○	○
j)	An asbestos related illness	○	○	○	○	○
k)	Arthritis	○	○	○	○	○

Q70 Please describe any other serious illnesses you have had within the last 2 years.

Q71 Have any of your blood relatives (brothers, sisters, parents or children) had the illnesses listed below? (**Please mark all that apply**):

		**Yes**	**No**
a)	Asthma	○	○
b)	Bronchitis	○	○
c)	Heart disease or Angina	○	○
d)	Lung cancer	○	○
e)	Tuberculosis (TB)	○	○

Section 14 - Other changes in your health

Q72 Please describe any changes in your health or anything unusual or different about your body and health, you have noticed during the last 2 years.

Q73 Please describe any medication, or illnesses not mentioned above, which might have caused your symptoms:

Section 15 - Smoking History

Q74 Have you ever smoked?
(Smoking is defined as smoking one cigarette/pipe/cigar a day for as long as one year.)

**Yes**		**No**	
○	**Please go to question 75**	○	**End of questionnaire**

Q75 When did you start smoking? Year ---------------

Q76 What is the total number of years in your life that you have smoked? ____________

Q77 Do you currently smoke?

Yes ○ Q77 On average (over your lifetime), how much do you smoke on a week day?
____________ cigarettes
____________ cigars
oz ____________ tobacco

No ○

Q78 When did you give up smoking altogether? Year ---------------

Q79 On average, how much did you used to smoke on a week day?
____________ cigarettes
____________ cigars
oz ____________ tobacco

Thank you very much for your time.

Please return this questionnaire to: either the **SPUtNIk** research team at your scan appointment or post to Lucy Brindle in the **prepaid envelope provided ONLY** after the research team have added the SPUtNIk code at the front.

If you have any concerns or questions about the IPCARD SPUtNIk Study please contact the Chief Investigator for the IPCARD SPUtNIk (questionnaire) Study, Dr Lucy Brindle, at the Faculty of Health Sciences, University of Southampton by calling 023 8059 8526 or email L.A.Brindle@soton.ac.uk
